# Over half of breakpoints in gene pairs involved in cancer-specific recurrent translocations are mapped to human chromosomal fragile sites

**DOI:** 10.1186/1471-2164-10-59

**Published:** 2009-01-30

**Authors:** Allison A Burrow, Laura E Williams, Levi CT Pierce, Yuh-Hwa Wang

**Affiliations:** 1 Department of Biochemistry, Wake Forest University School of Medicine, Medical Center Boulevard, Winston-Salem, NC 27157-1016, USA; 2 Department of Electrical Engineering and Computer Science, University of Kansas, Lawrence, KS 66045-7612, USA

## Abstract

**Background:**

Gene rearrangements such as chromosomal translocations have been shown to contribute to cancer development. Human chromosomal fragile sites are regions of the genome especially prone to breakage, and have been implicated in various chromosome abnormalities found in cancer. However, there has been no comprehensive and quantitative examination of the location of fragile sites in relation to all chromosomal aberrations.

**Results:**

Using up-to-date databases containing all cancer-specific recurrent translocations, we have examined 444 unique pairs of genes involved in these translocations to determine the correlation of translocation breakpoints and fragile sites in the gene pairs. We found that over half (52%) of translocation breakpoints in at least one gene of these gene pairs are mapped to fragile sites. Among these, we examined the DNA sequences within and flanking three randomly selected pairs of translocation-prone genes, and found that they exhibit characteristic features of fragile DNA, with frequent AT-rich flexibility islands and the potential of forming highly stable secondary structures.

**Conclusion:**

Our study is the first to examine gene pairs involved in all recurrent chromosomal translocations observed in tumor cells, and to correlate the location of more than half of breakpoints to positions of known fragile sites. These results provide strong evidence to support a causative role for fragile sites in the generation of cancer-specific chromosomal rearrangements.

## Background

Tumor cells exhibit various forms of genomic instability, including chromosomal rearrangements, many of which directly contribute to the neoplastic process rather than occurring as a consequence [1,2]. Rearrangements causing the deletion, insertion or translocation of genetic material often result in the expression of altered gene products with oncogenic potential, or the loss of tumor suppressive functions. Although the mechanisms of these processes remain elusive, it is evident that DNA breakage is an initiating event.

There are a variety of ways by which a cell acquires DNA breaks. Breaks can arise from any agent that affects the primary structure of the double helix, like endogenous reactive oxygen species or exogenous factors such as ionizing radiation [3]. More recent reports suggest that, in addition, regions of the genome especially susceptible to breakage termed "fragile sites" may also cause DNA strand breakage. One study using chromosome banding has provided compelling evidence supporting a role for fragile sites in cancer development by demonstrating a significant association between sites of chromosome rearrangements found in tumor cells and fragile sites [4]. Therefore, it has been proposed that fragile sites may contribute to the genetic instability observed in cancer cells [5], but a direct role has not yet been proven.

Fragile sites are defined as non-random chromosomal loci that exhibit gaps and breaks on metaphase chromosomes under conditions which partially inhibit DNA synthesis [6]. Fragile sites are classified as common or rare, depending on their frequency in the population, and are further divided according to their mode of induction in cultured cells. Common fragile sites are present in all individuals, and are therefore believed to represent a normal component of chromosome structure [7]. In contrast, rare fragile sites are found in less than 5% of the population, and are inherited in a Mendelian manner [8,9]. To date, about 121 different fragile sites have been identified, but the number may increase. The majority of fragile sites can be induced by environmental agents and chemicals, including caffeine, alcohol and cigarette smoke [6]. Variability of fragile site breakage has been observed within individuals [10], which may reflect exposure to such factors, with high levels being associated with cancer [11]. Many genes located within or spanning these sites have been identified as tumor suppressors or oncogenes, and fragile sites have been found to be preferential targets of environmental mutagens and carcinogens [12]. Numerous studies have also revealed a significant association between the location of fragile sites and sites of a limited number of chromosome defects in cancer cells [13-15]. Moreover, Durkin *et al. *demonstrated that the deletions of the type seen in cancer can be produced within fragile site FRA3B, a site of rearrangements and deletions that are among the most common type of aberrations found in tumors [16], further supporting a role for fragile sites in cancer development.

Although the mechanisms of fragile site breakage are still unclear, several factors have been identified which contribute to fragile site instability. Studies have shown that a deficiency of proteins in the ATR-dependent cell cycle checkpoint pathway dramatically increases fragile site breakage [14]. In addition, all fragile DNA sequences examined so far demonstrate significantly high flexibility [17], and are comprised of AT-rich flexibility islands that can readily fold into stable secondary structures capable of perturbing DNA replication [17,18]. Moreover, all fragile sites studied to date, including FRAXA [19], FRA7H [20], FRA3B [21], FRA10B [22], and FRA16B [22] have been identified as late-replicating regions of the genome.

It is not entirely clear why fragile sites are susceptible to delayed replication, but it has been proposed that the flexible, AT-rich DNA sequences cause the replication fork to pause or stall at sites of secondary structure formation [23]. Supporting this hypothesis, Zhang and Freudenreich demonstrated that a polymorphic AT-dinucleotide repeat capable of forming a cruciform structure within fragile site FRA16D stalls replication fork progression in yeast, leading to increased chromosome breakage [24]. Collectively, these results suggest a shared molecular basis among fragile sites, indicating that the DNA sequences within these regions can present significant difficulties to the replication machinery, and require the activation of DNA damage checkpoint proteins. Fragile sites are therefore believed to represent unreplicated regions of the genome that have escaped the replication checkpoint, and are visible as gaps and breaks on metaphase chromosomes [25].

Although strong correlations have been made between the sites of breakage in a limited number of chromosome rearrangements and the position of fragile sites, there has been no systematic demonstration of fragile site locations relative to breakpoints in all known chromosome aberrations. Due to the availability of extensive databases containing chromosome abnormalities found in various types of tumors, and the increasing number of fragile sites being mapped, we have compiled a table of recurrent translocations in which each participating gene set generates cancer-specific fusion transcripts, and mapped their breakpoints to fragile sites. We found that in more than half (52%) of the translocation-participating gene sets, breakpoints within either one or both genes are located at fragile sites. These results suggest that chromosome fragility, particularly at fragile sites, may contribute to the generation of fusion transcripts in cancer cells. Furthermore, we have analyzed the DNA sequences at and around translocation-prone genes mapped to fragile sites for helix flexibility and the potential to form secondary structures. Our results demonstrate that the DNA sequences contain frequent AT-rich flexibility islands, and are capable of forming highly stable secondary structures, supporting their propensity for breakage.

## Results

### Breakpoints in over half (52%) of gene pairs involved in cancer-specific recurrent translocations are mapped to fragile sites

To comprehensively investigate the relationship between fragile sites and translocation breakpoints found in cancer, we examined all chromosome defects associated with various types of tumors, and identified recurrent translocations in which translocation breakpoints in either one or both participating genes co-localize with a fragile site. Taking advantage of the comprehensive databases already available, we examined a total of 444 different sets of translocation-participating genes involved in 451 translocations obtained from the "Translocation breakpoints In Cancer database" (TICdb) [26] and Mitelman Database of Chromosome Aberrations in Cancer [27]. We identified all genes involved in translocations that mapped to fragile sites, and compared the position of each breakpoint to fragile sites (Additional file 1). The types of fragile sites, rare or common, were also documented in Additional file 1. We found that 52 (12%) translocation-participating gene pairs have breakpoints for which both genes co-localize with fragile sites (Table 1), and 177 (40%) gene sets contain one gene for which the translocation breakpoint is located at a fragile site (Additional file 2). Therefore, we concluded that a significant number (52%) of gene pairs involved in cancer-specific recurrent translocations have at least one gene mapped to fragile sites. The majority (65%) of translocation breakpoints are located at common fragile sites, as opposed to rare sites. In Table 1, the partner genes in each translocation-participating pair are specified as 5' or 3' for the position in which they appear in the cancer-specific fusion transcript, and the types of cancer for each gene pair are listed. Interestingly, the table shows that some genes involved in translocations, like *MLL *and *EWSR1*, are most often found at the 5' end of the fusion product. Our data also indicate that fragile sites are involved in the abnormalities seen in a variety of cancers including leukemias, lymphomas, and other solid tumors, such as those of thyroid, breast, and lung.

**Table 1:** Translocation breakpoints mapped to fragile sites in both partner genes of recurrent cancer-specific translocations

	**5'-^b^**		**3'-^b^**		
**Translocation^a^**	**Gene**	**Fragile Site**	**Gene**	**Fragile Site**	**Cancer^c^**

t(2;18)(p11;q21)	BCL2	FRA18B	IGK@	FRA2L	Diffuse large B-cell lymphoma

t(16;16)(p13;q22), inv(16)(p13q22)	CBFB	FRA16B, FRA16C	MYH11	FRA16A	Acute myeloid leukemia

inv(10)(q11q21)	CCDC6	FRA10C	RET	FRA10G	Papillary thyroid carcinoma

t(11;19)(q13;p13)	CCND1	FRA11H	FSTL3	FRA19B	Chronic lymphocytic leukemia

t(2;7)(p11;q21)	CDK6	FRA7E	IGK@	FRA2L	B-cell lymphoma, Chronic lymphocytic leukemia

t(7;11)(q21;q23)	CDK6	FRA7E	MLL	FRA11B, FRA11G	Acute lymphoblastic leukemia

t(5;7)(q35;q21)	CDK6	FRA7E	TLX3	FRA5G	Acute lymphoblastic leukemia

t(12;22)(q13;q12)	EWSR1	FRA22B	ATF1	FRA12A	Soft tissue tumor

t(2;22)(q33;q12)	EWSR1	FRA22B	CREB1	FRA2I	Angiomatoid fibrous histiocytoma

inv(22)(q12q12)	EWSR1	FRA22B	PATZ1	FRA22B	Small round cell tumor

t(6;22)(p21;q12)	EWSR1	FRA22B	POU5F1	FRA6H	Undifferentiated bone tumor

t(2;22)(q31;q12)	EWSR1	FRA22B	SP3	FRA2G	Ewing tumor/small round cell tumor

t(11;22)(p13;q12)	EWSR1	FRA22B	WT1	FRA11E	Soft tissue tumor

del(4)(q12q12)^d^	FIP1L1	FRA4B	PDGFRA	FRA4B	Hypereosinophilic syndrome

inv(6)(p21q21)	HMGA1	FRA6H	LAMA4	FRA6F	Pulmonary chondroid hamartoma

t(3;6)(q27;p21)	HSP90AB1	FRA6H	BCL6	FRA3C	B-cell tumors

t(1;2)(p22;p11)	IGK@	FRA2L	BCL10	FRA1D	B-cell lymphoma

t(2;19)(p11;q13)	IGK@	FRA2L	BCL3	FRA19A	Mature B-cell neoplasm

t(2;3)(p11;q27)	IGK@	FRA2L	BCL6	FRA3C	Mature B-cell neoplasm, Follicular lymphoma

t(2;11)(p11;q13)	IGK@	FRA2L	CCND1	FRA11A, FRA11H	Mature B-cell neoplasm

t(2;18)(p11;q21)	IGK@	FRA2L	FVT1	FRA18B	Follicular lymphoma

t(3;16)(q27;p12)	IL21R	FRA16E	BCL6	FRA3C	Diffuse large B-cell lymphoma

t(11;19)(q13;q13.4)	MALAT1	FRA11H	MHLB1	FRA19A	Undifferentiated embryonal sarcoma

t(6;11)(p21.1;q13)	MALAT1	FRA11H	TFEB	FRA6H	Pediatric renal neoplasm

t(4;11)(q21.3-22.1;q23)	MLL	FRA11B, FRA11G	AFF1	FRA4F	Acute lymphoblastic leukemia, Acute myeloid leukemia

t(2;11)(q11;q23)	MLL	FRA11B, FRA11G	AFF3	FRA2A	Acute lymphoblastic leukemia

t(5;11)(q31;q23)	MLL	FRA11B, FRA11G	AFF4	FRA5C	Acute lymphoblastic leukemia

del(11)(q23q23)^d^	MLL	FRA11B, FRA11G	ARHGEF12	FRA11B, FRA11G	Acute myeloid leukemia

t(11;11)(q13;q23)	MLL	FRA11B, FRA11G	ARHGEF17	FRA11H	Acute myeloid leukemia

del(11)(q23q23)^d^	MLL	FRA11B, FRA11G	CBL	FRA11B, FRA11G	Acute myeloid leukemia

t(11;19)(q23;p13)	MLL	FRA11B, FRA11G	ELL	FRA19B	Acute myeloid leukemia

t(11;22)(q23;q13)	MLL	FRA11B, FRA11G	EP300	FRA22A	Acute myeloid leukemia

t(1;11)(p32;q23)	MLL	FRA11B, FRA11G	EPS15	FRA1B	Acute myeloid leukemia

t(6;11)(q21;q23)	MLL	FRA11B, FRA11G	FOXO3A	FRA6F	Acute myeloid leukemia

t(3;11)(q25;q23)	MLL	FRA11B, FRA11G	GMPS	FRA3D	Acute myeloid leukemia

t(11;14)(q23;q23)	MLL	FRA11B, FRA11G	GPHN	FRA14B	Acute myeloid leukemia

t(11;19)(q23;p13.3)	MLL	FRA11B, FRA11G	MLLT1	FRA19B	Acute myeloid leukemia

t(1;11)(q21;q23)	MLL	FRA11B, FRA11G	MLLT11	FRA1F	Acute myeloid leukemia

t(9;11)(p21;q23)	MLL	FRA11B, FRA11G	MLLT3	FRA9A, FRA9C	Acute myeloid leukemia

t(11;19)(q23;p13)	MLL	FRA11B, FRA11G	MYO1F	FRA19B	Acute myeloid leukemia

inv(11)(q14q23)	MLL	FRA11B, FRA11G	PICALM	FRA11F	Acute myeloid leukemia

t(2;11)(q37;q23)	MLL	FRA11B, FRA11G	SEPT2	FRA2J	Acute myeloid leukemia

t(11;19)(q23;p13)	MLL	FRA11B, FRA11G	SH3GL1	FRA19B	Acute myeloid leukemia

t(6;11)(q13;q23)	MLL	FRA11B, FRA11G	SMAP1	FRA6D	Acute myeloid leukemia

t(10;11)(q21;q23)	MLL	FRA11B, FRA11G	TET1	FRA10C	Acute myeloid leukemia

t(9;9)(p21;p21)	MTS2	FRA9A, FRA9C	MTS1	FRA9A, FRA9C	Acute lymphoblastic leukemia

inv(10)(q11q11)	NCOA4	FRA10G	RET	FRA10G	Papillary thyroid carcinoma

t(3;5)(q25;q35)	NPM1	FRA5G	MLF1	FRA3D	Acute myeloid leukemia

t(3;6)(q27;p21)	PIM1	FRA6H	BCL6	FRA3C	Diffuse large B-cell lymphoma

t(3;6)(q27;p21)	SFRS3	FRA6H	BCL6	FRA3C	Follicular lymphoma

t(19;19)(p13;q13)	TCF3	FRA19B	TFPT	FRA19A	Acute lymphoblastic leukemia

inv(1)(q21q31)	TPM3	FRA1F	TPR	FRA1K	Papillary thyroid carcinoma

^a^The translocation names for each unique gene set are indicated.

^b^Position in which the gene appears in the cancer-specific fusion transcript

^c^For a complete description, see the Mitelman database of Chromosome Aberrations in Cancer [27].

^d^Deletions creating a fusion between two genes

### Translocation-prone genes exhibit characteristics of fragile sites

**Figure 1:**
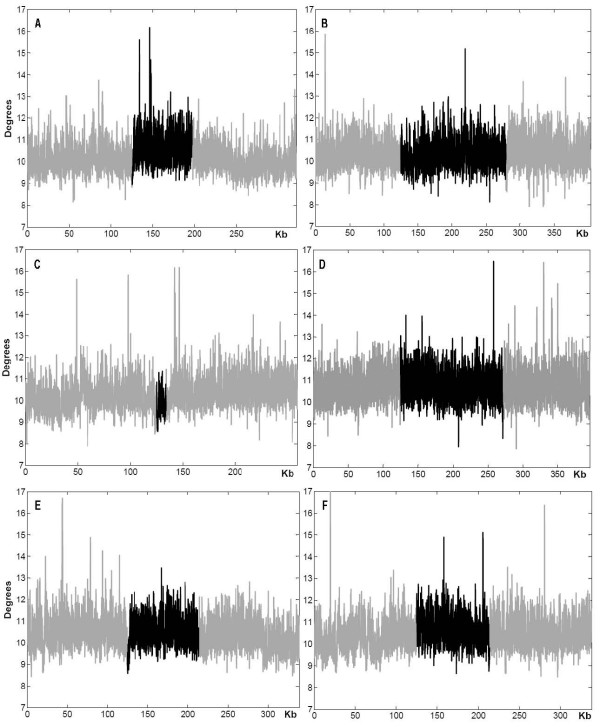
**DNA flexibility analysis of translocation-prone and fragile site co-localized genes**. DNA sequences within and flanking genes (A) *CBFB *(B) *MYH11 *(C) *HMGA1 *(D) *LAMA4 *(E) *MLL *(F) *AFF4 *were analyzed using the FlexStab program. The analysis was performed over the length of the entire gene (shaded in black) plus 125 kb flanking on each side (shaded in gray). The *x *axis indicates the size of the analyzed sequences, and the *y *axis shows degrees of inclination in the twist angle. Windows with values > 13.7° were considered as significantly high flexibility peaks [17].

**Figure 2:**
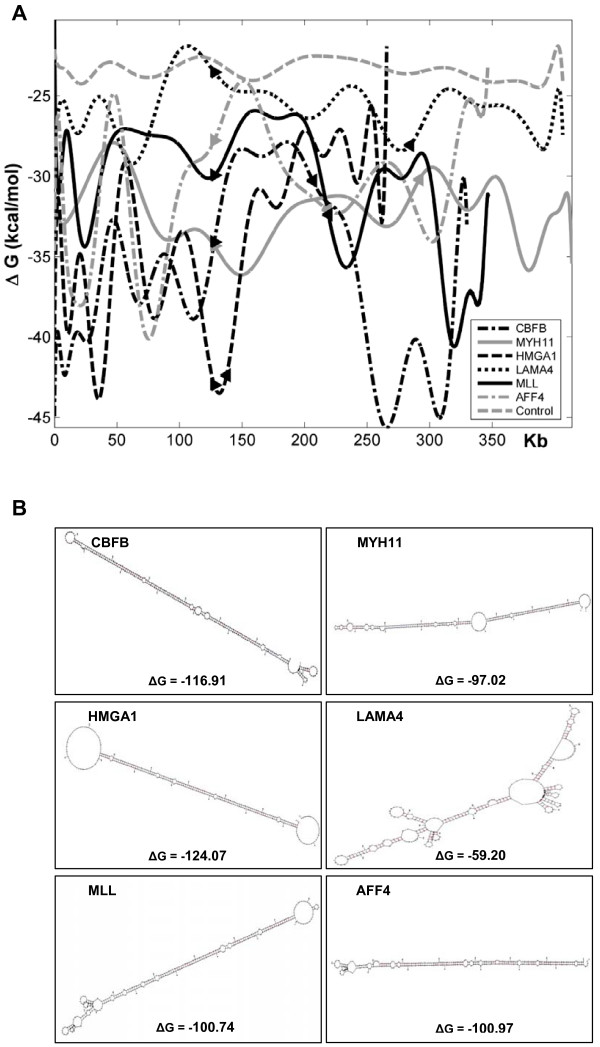
**Secondary structure analysis of *CBFB, MYH11, HMGA1, LAMA4, MLL, and AFF4 *loci**. (A) Comparison of potential to form secondary structure for these genes versus a control. The computed lowest free energy of predicted DNA secondary structures from segments of 300 nt in length, overlapping in 150 nt steps, has been fit to a curve for each gene. The Matlab function *polyfit *finds coefficients of a polynomial P(X) of degree N that fit the raw data best in a least-squares sense. The analysis was performed over the length of the entire gene plus 125 kb flanking on each side. The arrows indicate where a gene begins and ends. The control sequence was generated by randomizing *LAMA4 *1000 times. The *x *axis indicates the size of the analyzed sequences, and the *y *axis displays the free energy of the predicted structure. Raw data plots for each gene are included in Additional file 3. (B) The most stable structure predicted for each gene, as produced by MFOLD. Each structure represents the 300 nt segment with the lowest ΔG value.

Fragile sites have been shown to contain intrinsic features within the DNA sequence that confer a predisposition to fragility [28]. Most fragile sites sequenced to date contain highly flexible sequences and AT-rich islands, with the potential to form secondary structures, which are significantly more stable than same-length random DNA sequences [17,18]. Therefore, to determine whether genes involved in cancer-specific recurrent translocations exhibit properties of fragile sites, we analyzed three gene pairs from Table 1 (*CBFB/MYH11*, *HMGA1/LAMA4*, and *MLL/AFF4*), and their flanking sequences, for flexibility, A/T content, and the propensity to form stable secondary structures. Using the FlexStab program, we found that, with the exception of the *MYH11 *locus (Figure 1), DNA sequences with significantly high flexibility occur more frequently within the regions harboring translocation-prone genes than is predicted for control DNA [17]. The control DNA, from various regions of the human genome where no fragile sites were identified, contains approximately one high-flexibility peak every 100 kb [17]. Further sequence composition analysis demonstrated that the sequences within the flexibility peaks consist of a very high A/T content (Table 2), similar to what was previously reported for fragile site regions (78% ± 1.4%), which is significantly different from that of nonflexible sequences (61% ± 3.6%) (P < 0.001) [18]. In addition, these sequences are rich in AT-dinucleotides (Table 2) to the same extent as found in the flexible peaks of fragile site regions (21% ± 0.5%), as compared to nonflexible DNA (8% ± 1%) [18]. Further, the secondary structure prediction analysis showed that the sequences within and surrounding genes participating in cancer-specific translocations can readily form highly stable secondary structures, as indicated by their ΔG values (Table 2) and predicted structures (Figure 2 and Additional file 3). Overall, our results demonstrate that the three gene pairs examined display characteristics of fragile DNA, which could lead to DNA breakage, supporting the notion that fragile sites may participate in the generation of chromosome rearrangements.

**Table 2:** Computational analysis of genes involved in cancer-specific recurrent translocations reveals characteristics of chromosomal fragile sites

	**FlexStab**			**MFOLD**
**Gene**	**Number of flexibility peaks/Kb**	**% A/T**	**% AT-dinucleotides**	**Lowest ΔG value^a ^(kcal/mol)**

CBFB	4/322	79 ± 3.9	24 ± 3.0	-116.91

MYH11	4/404	78 ± 5.5	23 ± 5.9	-97.02

HMGA1	6/259	81 ± 7.8	24 ± 3.6	-124.07

LAMA4	9/397	78 ± 2.7	23 ± 3.0	-59.2

MLL	5/339	78 ± 10.2	23 ± 4.8	-100.74

AFF4	4/338	81 ± 3.3	26 ± 3.1	-100.97

Fragile site		78 ± 1.4^c^	21 ± 0.5^c^	

Control	1/100^b^	61 ± 3.6^c^	8 ± 1.0^c^	-41.79^d^

^a^Lowest ΔG value, predicted by MFOLD

^b^Mishmar et al. [17] examined 1.1 Mb of non-fragile DNA, and showed that regions with significantly high flexibility occur every ~100 kb.

^c^Zlotorynski et al. [18]

^d^The LAMA4 sequence was randomized 1000 times to serve as a control, and then analyzed by MFOLD.

## Discussion

Research on understanding the significance of human chromosomal fragile sites in cancer has been very active. Fragile sites are most commonly associated with deletion breakpoints in tumor cells, while few translocations involving these sites have been reported. A limited number of translocation breakpoints have been reported near fragile sites, suggesting that chromosome fragility at these sites may contribute to these rearrangements [29]. Our study herein provides a comprehensive survey of all cancer-specific translocations to date. Therefore, by demonstrating that breakpoints in over half (52%) of the gene pairs co-map to fragile sites, these results provide strong evidence to support a role for fragile sites in the generation of cancer-specific translocations. It is important to note that we have chosen to focus on translocations and deletions leading to fusion transcripts, and have not included other types of rearrangements such as single gene deletions, insertions, or complex translocations. We have found that many of the genes examined in this study are commonly involved in these other types of rearrangements, like deletion of the FHIT gene located within fragile site FRA3B, and in some cases, the same set of genes is involved in multiple translocations observed in a variety of cancers. An interesting observation was that some genes located near fragile sites, such as *NUP98 *(11p15.4) which participates in twenty-two different translocations examined, could not be included in Additional file 1, because the gene was not located directly at a fragile site. Recently described fragile sites, like FRA6H at 6p21 [30], have been identified after discovering an association between the chromosome location and sites of recurrent aberrations in disorders. This suggests that 11p15.4 could be a fragile site that has not yet been identified, or that the proximal fragile sites FRA11C and FRA11I at 11p15.1 are larger than previously determined. Therefore, it is appropriate to assume that the total number of chromosomal aberrations in cancer associated with fragile sites could be even greater than presented in this study, arguing for the significance of the involvement of fragile sites in tumorigenesis.

In addition to solidifying the role of fragile sites participating in cancer development, this study also supports the common hypothesis for the molecular basis of fragility at these sites. We have shown that the DNA sequences within and surrounding three pairs of translocation-prone genes exhibit features of fragility. On average, peaks of significantly high flexibility occur more often than in random DNA, which is consistent with previous results [17]. We also found these peaks to have a high A/T content and to be rich in AT-dinucleotides to the same extent as established in fragile sites [18]. Furthermore, our data from the MFOLD program indicate that the sequences have the potential to form highly stable secondary structures, another distinct characteristic of fragile sites [18], which could disturb progression of the replication fork. Based on our results, and the proposed mechanism of fragile site expression, it is likely that the AT-rich flexibility islands within or flanking translocation-prone genes are able to stall replication by the formation of secondary structures, which may then lead to DNA strand breakage, and ultimately to chromosome rearrangements.

Several proteins involved in the replication checkpoint pathway are essential for maintaining stability at fragile sites [14]. These include the S phase and G_2_/M checkpoint kinase ATR [25], and its downstream targets BRCA1 [31], FACD2 [32], and CHK1 [33]. ATR is a major component of the checkpoint pathway, where it functions by sensing and responding to DNA damage, including stalled and collapsed replication forks [34,35]. It is hypothesized that ATR maintains fragile site stability by sensing and binding to single-stranded DNA resulting from stalled replication forks [25]. However, in the absence of ATR, the main transducer of the DNA double-strand break (DSB) signal, which is ATM, has been shown to regulate fragile site stability [36], indicating that DSBs also occur at fragile sites. Following breakage, chromosome rearrangements may take place via the homologous recombination (HR), nonhomologous end-joining (NHEJ) DSB repair pathways [37,38], or microhomology-mediated single-strand annealing (SSA) [39]. The repair of lesions at fragile sites is still not clear, but evidence suggests that all three pathways may be involved. Based on recent observations made by Lieber *et al*., it is hypothesized that the sequence-specific RAG complex involved in V(D)J recombination, which is an important process of the NHEJ type, may also recognize and cleave at non-B DNA structures [40], a feature shared by all fragile sites examined to date. Schwartz *et al*. [41] have shown that induction of fragile sites leads to RAD51 focus formation, and phosphorylation of DNA-PKcs, key components of the HR and NHEJ pathways, respectively. Furthermore, they found that down-regulation of RAD51 and DNA-PKcs increases fragile site instability, suggesting that both HR and NHEJ DSB repair pathways mediate break repair at fragile sites. In addition, the majority of breakpoints in a papillary thyroid carcinoma rearrangement mapped to fragile sites occur at microhomology patches, indicating that fragile site-associated rearrangements can also arise by microhomology-mediated SSA [42]. Although the underlying basis of chromosome rearrangements is still unclear, our results show that 39% of the translocations examined have only one breakpoint mapped to a fragile site, suggesting that gene rearrangements could be achieved with strand breakage at only one gene, as well as at both participating genes. To support this hypothesis, additional studies are needed to obtain a greater understanding of the mechanisms of chromosome rearrangements.

The most intriguing finding from this study is that the majority (65%) of fragile sites mapped to translocation breakpoints are common fragile sites, which are present in all individuals, and can be induced by a variety of environmental factors and chemical agents. Interestingly, most cancers associated with the translocations examined in this study have little or no genetic component. These observations suggest that exposure to fragile site-inducing chemicals and/or reduced levels of proteins critical for the maintenance of fragile sites may confer a risk for cancer-specific rearrangements. It will be important to identify factors that contribute to chromosome fragility, such as DNA sequences, proteins and environmental/dietary agents, since fragile sites are sensitive to a range of chemicals. Understanding the molecular basis of fragile sites could therefore allow development of a prognostic assay for cancer risk.

## Conclusion

Our study is the first to comprehensively compare the location of cancer-specific recurrent translocation breakpoints and fragile sites. We showed that breakpoints in over half (52%) of the translocation-participating gene pairs co-map to positions of known human chromosomal fragile sites. Furthermore, we demonstrated that the DNA sequences at and surrounding three pairs of translocation-prone genes that are mapped to fragile sites, exhibit frequent AT-rich flexibility islands and are capable of forming highly stable secondary structures, both of which are characteristics of fragile DNA. Thus, we have provided a greater understanding of the contribution of fragile sites in the formation of chromosomal translocations, and further supported a role for fragile sites in cancer development.

## Methods

### Data collection

Two databases were examined encompassing all chromosome rearrangements found in cancer. The freely available TICdb was downloaded from  (v2.3 May 2008), which contains the breakpoints of cancer-specific reciprocal translocations mapped to over 300 different human genes [26]. In addition, we cross-examined the Mitelman database of Chromosome Aberrations in Cancer, a database relating chromosomal aberrations to tumor characteristics for recurrent translocations using the Molecular Biology Associations search tool [27]. From these databases, 444 different pairs of genes participating in cancer-specific recurrent translocations were obtained. In some cases, the same set of genes is involved in multiple types of translocations. All of these translocations result in one or two cancer-specific fusion genes. For each gene, the chromosomal locus was obtained from the UCSC Genome Bioinformatics website , and compared to the mapped positions of all known fragile sites ( using "fragile site" as a search term) [30,43-45]. All genes located at fragile sites are highlighted in Additional file 1. The translocation breakpoints of all genes highlighted in Additional file 1 were compared to fragile site positions, to identify translocations in which the site of breakage is co-localized with a fragile site at either one (Additional file 2) or both (Table 1) genes involved in the translocation.

### Flexibility analysis

DNA helix flexibility was assessed using FlexStab, a computer program designed by Mishmar *et al. *[17], which measures potential local variations in DNA between consecutive base pairs, and is expressed as fluctuations in the twist angle. The analysis was performed over the length of the entire gene plus 125 kb flanking on each side, in windows of 100 bp with 25 bp shift increments. Windows with values > 13.7° were considered as significantly high flexibility peaks [17].

Three gene pairs in which both genes are mapped to fragile sites (*CBFB/MYH11, HMGA1/LAMA4, and MLL/AFF4*) were used for flexibility and secondary structure analysis. The examined sequences for each gene are: *CBFB *[nucleotides (nt) 20552249~20874160 of GenBank: NT_010498.15], *MYH11 *[nt 6985071~7388966 of NT_010393.15], *HMGA1 *[nt 24937900~25197258 of NT_007592.14], *LAMA4 *[nt 16473563~16870257 of NT_025741.14], *MLL *[nt 21744621~22083352 of NT_033899.7], and *AFF4 *[nt 34501084~34839367 of NT_034772.5].

### Assessment of secondary structure

Using Zukers' MFOLD program [46], the potential of a single-stranded DNA to form stable secondary structure can be predicted along with its free-energy value. For a given gene, 300 nt segments with 150 nt shift increments were used as input for MFOLD. The length of 300 nt was chosen because it equals the Okazaki initiation zone of the DNA replication fork in mammalian cells, which possesses a single-stranded property during DNA replication [47,48]. The default [Na^+^], [Mg^2+^], and temperature used were 1.0 M, 0.0 M, and 37°C, respectively. The program Bioperl  was used to manipulate the sequences, and Matlab (MathWorks) was used to perform the analysis of the data.

## Authors' contributions

AB and LEW compiled and analyzed the databases. AB and LCTP carried out the flexibility and secondary structure analyses. AB and Y-HW wrote the paper. Y-HW designed and coordinated the study. All authors read and approved the final manuscript.

## Supplementary Material

Additional file 1 **Comprehensive list of gene pairs involved in cancer-specific recurrent translocations which result in fusion transcripts.** The translocation name(s) for each unique set of genes and the chromosomal locations of all genes are indicated. Genes which co-map to fragile sites are highlighted in gray, and the fragile site is specified. Click here for file

Additional file 2 **Gene pairs involved in cancer-specific recurrent translocations in which the breakpoint in one gene co-localizes with a fragile site. **The translocation name(s) for each unique gene set is indicated. The gene within each set located at a fragile site is highlighted in gray, and the cancer(s) in which the fusion transcript is found is included. Click here for file

Additional file 3 **The computed lowest free energy of predicted DNA secondary structures.** The flanking 125 kb regions are shaded in light gray, and the gene region is shaded in black. The black box indicates the location of the most stable structure found in the sequence. The black line is the curve which best fits the raw data. These curves were generated using the *polyfit *function of the Matlab program, and are presented in Figure 2A. Click here for file
