# Supplementary material for: Over half of breakpoints in gene pairs involved in cancer-specific recurrent translocations are mapped to human chromosomal fragile sites
Source: BMC Genomics. 2009 Jan 30;10:59. doi: 10.1186/1471-2164-10-59 (PMC2642838; doi:10.1186/1471-2164-10-59)
Supplement: Additional file 1 — Comprehensive list of gene pairs involved in cancer-specific recurrent translocations which result in fusion transcripts. The translocation name(s) for each unique set of genes and the chromosomal locations of all genes are indicated. Genes which co-map to fragile sites are highlighted in gray, and the fragile site is specified. [file 1471-2164-10-59-S1.pdf]

Additional file 1 - Comprehensive list of gene pairs involved in cancer-specific recurrent translocations which result in fusion transcripts						
**Translocation**	**Gene**	**Location**	**Fragile Site**	**Gene**	**Location**	**Fragile Site**
t(7;12)(p22;q13)	ACTB	7p22.1	FRA7B (common, apc)	GLI1	12q13.3	
inv(7)(q21q34)	AKAP9	7q21.2	FRA7E (common, apc)	BRAF	7q34	
t(X;17)(p11;q25)	ASPSCR1	17q25.3		TFE3	Xp11.23	
inv(2)(p23q35)	ATIC	2q35		ALK	2p23.2-p23.1	
t(17;20)(q23;q13)	BCAS4	20q13.13		BCAS3	17q23.2	
t(2;3)(p16;q26)	BCL11A	2p16.1		MDS1	3q26.2	
t(5;14)(q35;q32)	BCL11B	14q32.2		NKX2E	5q35.2	FRA5G (rare, folic acid)
t(5;14)(q35;q32)	BCL11B	14q32.2		TLX3	5q35.1	FRA5G (rare, folic acid)
inv(14)(q11q32)	BCL11B	14q32.2		TRD@	14q11.2	
t(14;18)(q32;q21)	BCL2	18q21.33	FRA18B (common, apc)	IGH@	14q32.33	
t(2;18)(p11;q21)	BCL2	18q21.33	FRA18B (common, apc)	IGK@	2p11.2	FRA2L (rare, folic acid)
t(18;22)(q21;q11)	BCL2	18q21.33	FRA18B (common, apc)	IGL@	22q11.22	
t(8;19)(q24;q13)	BCL3	19q13.31	FRA19A (common, 5-aza)	MYC	8q24.21	
t(3;16)(q27;p13)	BCL6	3q27.3	FRA3C (common, apc)	CIITA	16p13.13	
t(3;8)(q27;q24)	BCL6	3q27.3	FRA3C (common, apc)	MYC	8q24.21	
t(1;14)(q21;q32)	BCL9	1q21.1	FRA1F (common, apc)	IGH@	14q32.33	
t(1;22)(q21;q11)	BCL9	1q21.1	FRA1F (common, apc)	IGL@	22q11.22	
t(9;22)(q34;q11)	BCR	22q11.23		ABL1	9q34.12	
t(8;22)(p12;q11)	BCR	22q11.23		FGFR1	8p12	
t(9;22)(p24;q11)	BCR	22q11.23		JAK2	9p24.1	
t(4;22)(q12;q11)	BCR	22q11.23		PDGFRA	4q12	FRA4B (common, BrdU)
t(11;18)(q22;q21)	BIRC3	11q22.2		MALT1	18q21.32	FRA18B (common, apc)
t(X;11)(q21;q23)	BRWD3	Xq21.1		ARHGAP20	11q22.3-q23.1	
t(8;12)(q21;q22)	BTG1	12q21.33	FRA12B (common, apc)	MYC	8q24.21	
t(7;15)(p21;q21)	C15ORF21	15q21.1		ETV1	7p21.2	
t(3;3)(q21;q26)	C3ORF27	3q21.3		EVI1	3q26.2	
t(2;11)(p23;p15)	CARS	11p15.4		ALK	2p23.2-p23.1	
t(16;16)(p13;q22), inv(16)(p13q22)	CBFB	16q22.1	FRA16B (rare, dist A), FRA16C (common, apc)	MYH11	16p13.11	FRA16A (rare, folic acid)
t(5;10)(q33;q21)	CCDC6	10q21.2	FRA10C (common, BrdU)	PDGFRB	5q33.1	
inv(10)(q11q21)	CCDC6	10q21.2	FRA10C (common, BrdU)	RET	10q11.21	FRA10G (common, apc)
t(5;14)(q33;q32)	CCDC88C	14q32.12		PDGFRB	5q33.1	
t(11;19)(q13;p13)	CCND1	11q13 .2	FRA11H (common, apc)	FSTL3	19p13.3	FRA19B (rare, folic acid)
t(5;6)(q32-33;q22)	CD74	5q33.1		ROS1	6q22.2	
t(16;17)(q21;p13)	CDH11	16q21		USP6	17p13.2	
t(7;11)(q21;q23)	CDK6	7q21.2	FRA7E (common, apc)	MLL	11q23.3	FRA11B (rare, folic acid), FRA11G (common, apc)
t(5;7)(q35;q21)	CDK6	7q21.2	FRA7E (common, apc)	TLX3	5q35.1	FRA5G (rare, folic acid)
t(5;11)(q12;q23)	CENPK	5q12.3		MLL	11q23.3	FRA11B (rare, folic acid), FRA11G (common, apc)
t(8;9)(p12;q33)	CEP110	9q33.2		FGFR1	8p12	
t(4;12)(q12;p13)	CHIC2	4q12	FRA4B (common, BrdU)	ETV6	12p13.2	
t(4;19)(q35;q13)	CIC	19q13.2	FRA19A (common, 5-aza)	DUX4	4q35.2	
t(2;17)(p23;q23)	CLTC	17q23.1	FRA17B (common, apc)	ALK	2p23.2-p23.1	

t(X;17)(p11;q23)	CLTC	17q23.1	FRA17B (common, apc)	TFE3	Xp11.23	
t(2;22)(p23;q11)	CLTCL1	22q11.21		ALK	2p23.2-p23.1	
t(3;17)(q21;p13)	CNBP	3q21.3		USP6	17p13.2	
t(17;22)(q21;q13)	COL1A1	17q21.33		PDGFB	22q13.1	FRA22A (rare, folic acid)
t(17;17)(p13;q21)	COL1A1	17q21.33		USP6	17p13.2	
t(7;8)(q21;q12)	COL1A2	7q21.3		PLAG1	8q12.1	
t(X;6)(q22;q13-14)	COL4A5	Xq22.3		COL12A1	6q13-q14.1	FRA6D (common, BrdU)
t(1;2)(p13;q37)	COL6A3	2q37.3	FRA2J (common, apc)	CSF1	1p13.3	
t(8;12)(p12;q15)	CPSF6	12q15		FGFR1	8p12	
t(11;19)(q21;p13)	CRTC1	19p13.11	FRA19B (rare, folic acid)	MAML2	11q21	
t(11;15)(q21;q26)	CRTC3	15q26.1		MAML2	11q21	
t(3;8)(p22;q12)	CTNNB1	3p22.1		PLAG1	8q12.1	
t(3;9)(q27;p24)	DMRT1	9p24.3		BCL6	3q27.3	FRA3C (common, apc)
t(1;1)(p36;q41)	DUSP10	1q41		PRDM16	1p36.32	FRA1A (common, apc)
t(5;12)(q33;q14)	EBF1	5q33.3		LOC204010	12q14.3	
t(X;21)(q25;q22)	ELF4	Xq25		ERG	21q22.2	
t(9;14)(q34;q32)	EML1	14q32.2		ABL1	9q34.12	
inv(2)(p21p23), del(2)(p21p23)*	EML4	2p21		ALK	2p23.2-p23.1	
t(5;12)(q33;p13)	ERC1	12p13.33		PDGFRB	5q33.1	
t(10;12)(q11;p13)	ERC1	12p13.33		RET	10q11.21	FRA10G (common, apc)
t(9;12)(q34;p13)	ETV6	12p13.2		ABL1	9q34.12	
t(1;12)(q25;p13)	ETV6	12p13.2		ABL2	1q25.2	
t(5;12)(q31;p13)	ETV6	12p13.2		ACSL6	5q31.1	FRA5C (common, apc)
t(1;12)(q21;p13)	ETV6	12p13.2		ARNT	1q21.2	FRA1F (common, apc)
t(12;12)(p13;q13)	ETV6	12p13.2		BAZ2A	12q13.3	
t(12;13)(p13;q12)	ETV6	12p13.2		CDX2	13q12.2	
t(3;12)(q26;p13)	ETV6	12p13.2		EVI1	3q26.2	
t(4;12)(p16;p13)	ETV6	12p13.2		FGFR3	4p16.3	
t(12;13)(p13;q12)	ETV6	12p13.2		FLT3	13q12.2	
t(6;12)(q22;p13)	ETV6	12p13.2		FRK	6q22.1	
t(10;12)(q24;p13)	ETV6	12p13.2		GOT1	10q24.2	FRA10A (rare, folic acid)
t(9;12)(p24;p13)	ETV6	12p13.2		JAK2	9p24.1	
t(3;12)(q26;p13)	ETV6	12p13.2		MDS1	3q26.2	
t(1;12)(p36;p13)	ETV6	12p13.2		MDS2	1p36.11	FRA1A (common, apc)
t(12;15)(p13;q25)	ETV6	12p13.2		NTRK3	15q25.3	
t(4;12)(q12;p13)	ETV6	12p13.2		PDGFRA	4q12	FRA4B (common, BrdU)
t(5;12)(q33;p13)	ETV6	12p13.2		PDGFRB	5q33.1	
t(12;17)(p13;p13)	ETV6	12p13.2		PER1	17p13.1	
inv(12)(p13q15)	ETV6	12p13.2		PTPRR	12q15	
t(12;21)(p13;q22)	ETV6	12p13.2		RUNX1	21q22.12	
t(6;12)(q23;p13)	ETV6	12p13.2		STL	6q23	
t(9;12)(q22;p13)	ETV6	12p13.2		SYK	9q22.2	
t(12;22)(q13;q12)	EWSR1	22q12.2	FRA22B (common, apc)	ATF1	12q13.13	FRA12A (rare, folic acid)

t(2;22)(q33;q12)	EWSR1	22q12.2	FRA22B (common, apc)	CREB1	2q33.3	FRA2I (common,apc)
t(12;22)(q13;q12)	EWSR1	22q12.2	FRA22B (common, apc)	DDIT3	12q13.3	
t(21;22)(q22;q12)	EWSR1	22q12.2	FRA22B (common, apc)	ERG	21q22.2	
t(7;22)(p21;q12)	EWSR1	22q12.2	FRA22B (common, apc)	ETV1	7p21.2	
t(17;22)(q21;q12)	EWSR1	22q12.2	FRA22B (common, apc)	ETV4	17q21.31	
t(2;22)(q35;q12)	EWSR1	22q12.2	FRA22B (common, apc)	FEV	2q35	
t(11;22)(q24;q12)	EWSR1	22q12.2	FRA22B (common, apc)	FLI1	11q24.3	
t(9;22)(q31;q12)	EWSR1	22q12.2	FRA22B (common, apc)	NR4A3	9q31.1	
inv(22)(q12q12)	EWSR1	22q12.2	FRA22B (common, apc)	PATZ1	22q12.2	FRA22B (common, apc)
t(6;22)(p21;q12)	EWSR1	22q12.2	FRA22B (common, apc)	POU5F1	6p21.33	FRA6H (common, apc)
t(2;22)(q31;q12)	EWSR1	22q12.2	FRA22B (common, apc)	SP3	2q31.1	FRA2G (common, apc)
t(11;22)(p13;q12)	EWSR1	22q12.2	FRA22B (common, apc)	WT1	11p13	FRA11E (common, apc)
t(12;22)(p13;q12)	EWSR1	22q12.2	FRA22B (common, apc)	ZNF384	12p13.31	
t(5;7)(q31;q34)	FCHSD1	5q31.3		BRAF	7q34	
t(6;8)(q27;p12)	FGFR1OP	6q27		FGFR1	8p12	
del(4)(q12q12)*	FIP1L1	4q12	FRA4B (common, BrdU)	PDGFRA	4q12	FRA4B (common, BrdU)
t(4;17)(q12;q21)	FIP1L1	4q12	FRA4B (common, BrdU)	RARA	17q21.2	
t(2;13)(q36;q14)	FOXO1A	13q14.11		PAX3	2q36.1	
t(X;11)(q13;q23)	FOXO4	Xq13.1		MLL	11q23.3	FRA11B (rare, folic acid), FRA11G (common, apc)
t(12;16)(q13;p11)	FUS	16p11.2		ATF1	12q13.13	FRA12A (rare, folic acid)
t(11;16)(p11;p11)	FUS	16p11.2		CREB3L1	11p11.2	
t(7;16)(q34;p11)	FUS	16p11.2		CREB3L2	7q33-q34	
t(12;16)(q13;p11)	FUS	16p11.2		DDIT3	12q13.3	
t(16;21)(p11;q22)	FUS	16p11.2		ERG	21q22.2	
t(2;16)(q35;p11)	FUS	16p11.2		FEV	2q35	
t(3;12)(q27;p13)	GAPDH	12p13.31		BCL6	3q27.3	FRA3C (common, apc)
t(5;12)(q33;q24)	GIT2	12q24.11	FRA12E (common, apc)	PDGFRB	5q33.1	
t(10;14)(q11;q32)	GOLGA5	14q32.12		RET	10q11.21	FRA10G (common, apc)
del(6)(q21q22)*	GOPC	6q22.2		ROS1	6q22.2	
del(8)(q12q24)*	HAS2	8q24.13	FRA8C (common, apc), FRA8E (rare, dist A)	PLAG1	8q12.1	
t(8;19)(p12;q13)	HERV-K (LOC113386)	19q13.43	FRA19A (common, 5-aza)	FGFR1	8p12	
t(5;7)(q33;q11)	HIP1	7q11.23	FRA7J (common, apc)	PDGFRB	5q33.1	
t(3;6)(q27;p22)	HIST1H4I	6p22.1		BCL6	3q27.3	FRA3C (common, apc)
inv(6)(p21q21)	HMGA1	6p21.31	FRA6H (common, apc)	LAMA4	6q21	FRA6F (common, apc)
t(12;14)(q14;q11)	HMGA2	12q14.3		CCNB1IP1	14q11.2	
t(8;12)(q22;q14)	HMGA2	12q14.3		COX6C	8q22.2	
t(2;12)(q37;q14)	HMGA2	12q14.3		CXCR7	2q37.3	FRA2J (common, apc)
t(5;12)(q33;q14)	HMGA2	12q14.3		EBF1	5q33.3	
t(12;13)(q14;q13)	HMGA2	12q14.3		LHFP	13q13.3	
t(3;12)(q28;q14)	HMGA2	12q14.3		LPP	3q28	
t(9;12)(p23;q14)	HMGA2	12q14.3		NFIB	9p23-p22.3	
t(12;14)(q14;q24)	HMGA2	12q14.3		RAD51L1	14q24.1	FRA14C (common, apc)
t(7;7)(p15;p21)	HNRPA2B1	7p15.2		ETV1	7p21.2	

t(8;10)(p11;q11)	HOOK3	8p11.21		RET	10q11.21	FRA10G (common, apc)
t(6;16)(p21;q22)	HP	16q22.3		MRPS10	6p21.1	FRA6H (common, apc)
t(3;14)(q27;q32)	HSP90AA1	14q32.31		BCL6	3q27.3	FRA3C (common, apc)
t(3;6)(q27;p21)	HSP90AB1	6p21.1	FRA6H (common, apc)	BCL6	3q27.3	FRA3C (common, apc)
t(1;14)(p22;q32)	IGH@	14q32.33		BCL10	1p22.3	FRA1D (common, apc)
t(2;14)(p16;q32)	IGH@	14q32.33		BCL11A	2p16.1	
t(14;19)(q32;q13)	IGH@	14q32.33		BCL3	19q13.31	FRA19A (common, 5-aza)
t(3;14)(q27;q32)	IGH@	14q32.33		BCL6	3q27.3	FRA3C (common, apc)
t(14;15)(q32;q11-13)	IGH@	14q32.33		BCL8	15q11.2	
t(11;14)(q13;q32)	IGH@	14q32.33		CCND1	11q13.2	FRA11A (rare, folic acid), FRA11H (common, apc)
t(12;14)(p13;q32)	IGH@	14q32.33		CCND2	12p13.32	
t(6;14)(p21;q32)	IGH@	14q32.33		CCND3	6p21.1	FRA6H (common, apc)
t(7;14)(q21;q32)	IGH@	14q32.33		CDK6	7q21.2	FRA7E (common, apc)
t(14;19)(q32;q13)	IGH@	14q32.33		CEBPA	19q13.11	FRA19A (common, 5-aza)
t(14;20)(q32;q13)	IGH@	14q32.33		CEBPB	20q13.13	
t(8;14)(q11;q32)	IGH@	14q32.33		CEBPD	8q11.21	
t(14;14)(q11;q32)	IGH@	14q32.33		CEBPE	14q11.2	
t(14;19)(q32;q13)	IGH@	14q32.33		CEBPG	19q13.11	FRA19A (common, 5-aza)
t(12;14)(q23;q32)	IGH@	14q32.33		CHST11	12q23.3	
t(11;14)(q23;q32)	IGH@	14q32.33		DDX6	11q23.3	FRA11B (rare, folic acid), FRA11G (common, apc)
t(7;14)(q21;q32)	IGH@	14q32.33		ERVWE1	7q21.2	FRA7E (common, apc)
t(12;14)(p13;q32)	IGH@	14q32.33		ETV6	12p13.2	
t(1;14)(q23;q32)	IGH@	14q32.33		FCGR2B	1q23.3	
t(1;14)(q21;q32)	IGH@	14q32.33		FCRL4	1q23.1	
t(4;14)(p16;q32)	IGH@	14q32.33		FGFR3	4p16.3	
t(3;14)(p14;q32)	IGH@	14q32.33		FOXP1	3p14.1	
t(6;14)(p22;q32)	IGH@	14q32.33		ID4	6p22.3	
t(14;22)(q32;q11)	IGH@	14q32.33		IGL@	22q11.22	
t(5;14)(q31;q32)	IGH@	14q32.33		IL3	5q31.1	FRA5C (common, apc)
t(6;14)(p25;q32)	IGH@	14q32.33		IRF4	6p25.3	
t(1;14)(p35;q32)	IGH@	14q32.33		LAPTM5	1p35.2	
t(1;14)(q25;q32)	IGH@	14q32.33		LHX4	1q25.2	
t(14;16)(q32;q23)	IGH@	14q32.33		MAF	16q23.1	
t(14;20)(q32;q12)	IGH@	14q32.33		MAFB	20q12	
t(14;18)(q32;q21)	IGH@	14q32.33		MALT1	18q21.32	FRA18B (common, apc)
t(1;14)(q22;q32)	IGH@	14q32.33		MUC1	1q22	
t(8;14)(q24;q32)	IGH@	14q32.33		MYC	8q24.21	
t(10;14)(q24;q32)	IGH@	14q32.33		NFKB2	10q24.32	
t(11;14)(q23;q32)	IGH@	14q32.33		PAFAH1B2	11q23.3	FRA11B (rare, folic acid), FRA11G (common, apc)
t(9;14)(p13;q32)	IGH@	14q32.33		PAX5	9p13.2	
t(11;14)(q23;q32)	IGH@	14q32.33		PCSK7	11q23.3	FRA11B (rare, folic acid), FRA11G (common, apc)
t(4;14)(p14;q32)	IGH@	14q32.33		RHOH	4p14	
t(14;19)(q32;q13)	IGH@	14q32.33		SPIB	19q13.33	FRA19A (common, 5-aza)

t(14;14)(q11;q32), inv(14)(q11q32)	IGH@	14q32.33		TRA@	14q11.2	
inv(14)(q11q32)	IGH@	14q32.33		TRD@	14q11.2	
t(4;14)(p16;q32)	IGH@	14q32.33		WHSC1	4p16.3	
t(14;16)(q32;q23)	IGH@	14q32.33		WWOX	16q23.1	FRA16D (common, apc)
t(1;2)(p22;p11)	IGK@	2p11.2	FRA2L (rare, folic acid)	BCL10	1p22.3	FRA1D (common, apc)
t(2;19)(p11;q13)	IGK@	2p11.2	FRA2L (rare, folic acid)	BCL3	19q13.31	FRA19A (common, 5-aza)
t(2;3)(p11;q27)	IGK@	2p11.2	FRA2L (rare, folic acid)	BCL6	3q27.3	FRA3C (common, apc)
t(2;11)(p11;q13)	IGK@	2p11.2	FRA2L (rare, folic acid)	CCND1	11q13.2	FRA11A (rare, folic acid), FRA11H (common, apc)
t(2;12)(p11;p13)	IGK@	2p11.2	FRA2L (rare, folic acid)	CCND2	12p13.32	
t(2;7)(p11;q21)	IGK@	2p11.2	FRA2L (rare, folic acid)	CDK6	7q21.2	FRA7E (common, apc)
t(2;18)(p11;q21)	IGK@	2p11.2	FRA2L (rare, folic acid)	FVT1	18q21.33	FRA18B (common, apc)
t(2;8)(p11;q24)	IGK@	2p11.2	FRA2L (rare, folic acid)	MYC	8q24.21	
t(2;8)(p11;q24)	IGK@	2p11.2	FRA2L (rare, folic acid)	PVT1	8q24.21	
t(2;6)(p11;q25)	IGK@	2p11.2	FRA2L (rare, folic acid)	ZC3H12D	6q25.1	
t(19;22)(q13;q11)	IGL@	22q11.22-q11.23		BCL3	19q13.31	FRA19A (common, 5-aza)
t(3;22)(q27;q11)	IGL@	22q11.22-q11.23		BCL6	3q27.3	FRA3C (common, apc)
t(11;22)(q13;q11)	IGL@	22q11.22-q11.23		CCND1	11q13.2	FRA11A (rare, folic acid), FRA11H (common, apc)
t(12;22)(p13;q11)	IGL@	22q11.22-q11.23		CCND2	12p13.32	
t(6;22)(p21;q11)	IGL@	22q11.22-q11.23		CCND3	6p21.1	FRA6H (common, apc)
t(7;22)(q21;q11)	IGL@	22q11.22-q11.23		CDK6	7q21.2	FRA7E (common, apc)
t(16;22)(q23;q11)	IGL@	22q11.22-q11.23		MAF	16q23.1	
t(8;22)(q24;q11)	IGL@	22q11.22-q11.23		MYC	8q24.21	
t(8;22)(q24;q11)	IGL@	22q11.22-q11.23		PVT1	8q24.21	
t(2;22)(p16;q11)	IGL@	22q11.22-q11.23		REL	2p16.1	
t(16;22)(q23;q11)	IGL@	22q11.22-q11.23		WWOX	16q23.1	FRA16D (common, apc)
t(4;16)(q27;p13)	IL2	4q27		DEXI	16p13.13	
t(4;16)(q27;p13)	IL2	4q27		TNFRSF17	16p13.13	
t(3;16)(q27;p12)	IL21R	16p12.1	FRA16E (rare, dist A)	BCL6	3q27.3	FRA3C (common, apc)
t(5;9)(q33;q22)	ITK	5q33.3		SYK	9q22.2	
t(6;7)(p21;p15)	JAZF1	7p15.2-p15.1		PHF1	6p21.32	FRA6H (common, apc)
t(7;17)(p15;q11)	JAZF1	7p15.2-p15.1		SUZ12	17q11.2	
t(2;17)(p23;q25)	KIAA1618	17q25.3		ALK	2p23.2-p23.1	
t(4;10)(q12;p11)	KIF5B	10p11.22		PDGFRA	4q12	FRA4B (common, BrdU)
t(10;14)(q11;q22)	KTN1	14q22.3		RET	10q11.21	FRA10G (common, apc)
t(12;16)(p13;p13)	LAG3	12p13.31		MYH11	16p13.11	FRA16A (rare, folic acid)
t(1;7)(p35;q34)	LCK	1p35.1		TRB@	7q34	
t(3;13)(q27;q14)	LCP1	13q14.12		BCL6	3q27.3	FRA3C (common, apc)
t(5;8)(p13;q12)	LIFR	5p13.1	FRA5A (common, BrdU)	PLAG1	8q12.1	
del(3)(q27q28)*	LPP	3q28		BCL6	3q27.3	FRA3C (common, apc)
t(7;19)(q34;p13)	LYL1	19p13.13	FRA19B (rare, folic acid)	TRB@	7q34	
t(11;19)(q13;q13.4)	MALAT1	11q13.1	FRA11H (common, apc)	MHLB1	19q13.4	FRA19A (common, 5-aza)
t(6;11)(p21.1;q13)	MALAT1	11q13.1	FRA11H (common, apc)	TFEB	6p21.1	FRA6H (common, apc)
t(3;18)(p21;q21)	MALT1	18q21.32	FRA18B (common, apc)	MAP4	3p21.31	

t(1;19)(q23;p13)	MEF2D	1q22		DAZAP1	19p13.3	FRA19B (rare, folic acid)
t(10;11)(p12;q23)	MLL	11q23.3	FRA11B (rare, folic acid), FRA11G (common, apc)	ABI1	10p12.1	
t(11;17)(q23;q12)	MLL	11q23.3	FRA11B (rare, folic acid), FRA11G (common, apc)	ACACA	17q12	
t(4;11)(q21;q23)	MLL	11q23.3	FRA11B (rare, folic acid), FRA11G (common, apc)	AFF1	4q21.3-q22.1	FRA4F (common, apc)
t(2;11)(q11;q23)	MLL	11q23.3	FRA11B (rare, folic acid), FRA11G (common, apc)	AFF3	2q11.2	FRA2A (rare, folic acid)
t(5;11)(q31;q23)	MLL	11q23.3	FRA11B (rare, folic acid), FRA11G (common, apc)	AFF4	5q31.1	FRA5C (common, apc)
t(5;11)(q31;q23)	MLL	11q23.3	FRA11B (rare, folic acid), FRA11G (common, apc)	ARHGAP26	5q31.3	
del(11)(q23q23)*	MLL	11q23.3	FRA11B (rare, folic acid), FRA11G (common, apc)	ARHGEF12	11q23.3	FRA11B (rare, folic acid), FRA11G (common, apc)
t(11;11)(q13;q23)	MLL	11q23.3	FRA11B (rare, folic acid), FRA11G (common, apc)	ARHGEF17	11q13.4	FRA11H (common, apc)
t(11;15)(q23;q15)	MLL	11q23.3	FRA11B (rare, folic acid), FRA11G (common, apc)	CASC5	15q15.1	
del(11)(q23q23)*	MLL	11q23.3	FRA11B (rare, folic acid), FRA11G (common, apc)	CBL	11q23.3	FRA11B (rare, folic acid), FRA11G (common, apc)
t(11;12)(q23;q13)	MLL	11q23.3	FRA11B (rare, folic acid), FRA11G (common, apc)	CIP29	12q13.2	
t(11;16)(q23;p13.3)	MLL	11q23.3	FRA11B (rare, folic acid), FRA11G (common, apc)	CREBBP	16p13.3	
t(9;11)(q33;q23)	MLL	11q23.3	FRA11B (rare, folic acid), FRA11G (common, apc)	DAB2IP	9q33.2	
t(3;11)(q21;q23)	MLL	11q23.3	FRA11B (rare, folic acid), FRA11G (common, apc)	EEFSEC	3q21.3	
t(11;19)(q23;p13)	MLL	11q23.3	FRA11B (rare, folic acid), FRA11G (common, apc)	ELL	19p13.11	FRA19B (rare, folic acid)
t(11;22)(q23;q13)	MLL	11q23.3	FRA11B (rare, folic acid), FRA11G (common, apc)	EP300	22q13.2	FRA22A (rare, folic acid)
t(1;11)(p32;q23)	MLL	11q23.3	FRA11B (rare, folic acid), FRA11G (common, apc)	EPS15	1p32.3	FRA1B (common, apc)
t(6;11)(q21;q23)	MLL	11q23.3	FRA11B (rare, folic acid), FRA11G (common, apc)	FOXO3A	6q21	FRA6F (common, apc)
t(4;11)(p12;q23)	MLL	11q23.3	FRA11B (rare, folic acid), FRA11G (common, apc)	FRYL	4p12	
t(11;17)(q23;p13)	MLL	11q23.3	FRA11B (rare, folic acid), FRA11G (common, apc)	GAS7	17p13.1	
t(3;11)(q25;q23)	MLL	11q23.3	FRA11B (rare, folic acid), FRA11G (common, apc)	GMPS	3q25.31	FRA3D (common, apc)
t(11;14)(q23;q23)	MLL	11q23.3	FRA11B (rare, folic acid), FRA11G (common, apc)	GPHN	14q23.3	FRA14B (common, apc)
t(11;17)(q23;q12)	MLL	11q23.3	FRA11B (rare, folic acid), FRA11G (common, apc)	LASP1	17q12	
t(3;11)(q28;q23)	MLL	11q23.3	FRA11B (rare, folic acid), FRA11G (common, apc)	LPP	3q28	
inv(11)(q21q23)	MLL	11q23.3	FRA11B (rare, folic acid), FRA11G (common, apc)	MAML2	11q21	
t(11;20)(q23;q11)	MLL	11q23.3	FRA11B (rare, folic acid), FRA11G (common, apc)	MAPRE1	20q11.21	
t(11;19)(q23;p13.3)	MLL	11q23.3	FRA11B (rare, folic acid), FRA11G (common, apc)	MLLT1	19p13.3	FRA19B (rare, folic acid)
t(10;11)(p12;q23)	MLL	11q23.3	FRA11B (rare, folic acid), FRA11G (common, apc)	MLLT10	10p12.31	
t(1;11)(q21;q23)	MLL	11q23.3	FRA11B (rare, folic acid), FRA11G (common, apc)	MLLT11	1q21.2	FRA1F (common, apc)
t(9;11)(p21;q23)	MLL	11q23.3	FRA11B (rare, folic acid), FRA11G (common, apc)	MLLT3	9p21.3	FRA9A (rare, folic acid), FRA9C (common, BrdU)
t(6;11)(q27;q23)	MLL	11q23.3	FRA11B (rare, folic acid), FRA11G (common, apc)	MLLT4	6q27	
t(11;17)(q23;q12)	MLL	11q23.3	FRA11B (rare, folic acid), FRA11G (common, apc)	MLLT6	17q12	
t(11;19)(q23;p13)	MLL	11q23.3	FRA11B (rare, folic acid), FRA11G (common, apc)	MYO1F	19p13.2	FRA19B (rare, folic acid)
t(3;11)(p21;q23)	MLL	11q23.3	FRA11B (rare, folic acid), FRA11G (common, apc)	NCKIPSD	3p21.31	
inv(11)(q14q23)	MLL	11q23.3	FRA11B (rare, folic acid), FRA11G (common, apc)	PICALM	11q14.2	FRA11F (common, apc)
t(11;17)(q23;q21)	MLL	11q23.3	FRA11B (rare, folic acid), FRA11G (common, apc)	RARA	17q21.2	
t(4;11)(q21;q23)	MLL	11q23.3	FRA11B (rare, folic acid), FRA11G (common, apc)	SEPT11	4q21.1	
t(2;11)(q37;q23)	MLL	11q23.3	FRA11B (rare, folic acid), FRA11G (common, apc)	SEPT2	2q37.3	FRA2J (common, apc)
t(11;22)(q23;q11)	MLL	11q23.3	FRA11B (rare, folic acid), FRA11G (common, apc)	SEPT5	22q11.21	
t(X;11)(q24;q23)	MLL	11q23.3	FRA11B (rare, folic acid), FRA11G (common, apc)	SEPT6	Xq24	
t(11;17)(q23;q25)	MLL	11q23.3	FRA11B (rare, folic acid), FRA11G (common, apc)	SEPT9	17q25.2-q25.3	
t(11;19)(q23;p13)	MLL	11q23.3	FRA11B (rare, folic acid), FRA11G (common, apc)	SH3GL1	19p13.3	FRA19B (rare, folic acid)
t(6;11)(q13;q23)	MLL	11q23.3	FRA11B (rare, folic acid), FRA11G (common, apc)	SMAP1	6q13	FRA6D (common, BrdU)
t(4;11)(q35;q23)	MLL	11q23.3	FRA11B (rare, folic acid), FRA11G (common, apc)	SORBS2	4q35.1	
t(10;11)(q21;q23)	MLL	11q23.3	FRA11B (rare, folic acid), FRA11G (common, apc)	TET1	10q21.3	FRA10C (common, BrdU)
t(11;15)(q23;q15)	MLL	11q23.3	FRA11B (rare, folic acid), FRA11G (common, apc)	ZFYVE19	15q15.1	
t(12;22)(p13;q12)	MN1	22q12.1		ETV6	12p13.2	

t(7;12)(q36;p13)	MNX1	7q36.3	FRA7I (common, apc)	ETV6	12p13.2	
t(7;17)(p15;q22)	MSI2	17q22		HOXA9	7p15.2	
t(X;2)(q11;p23)	MSN	Xq11.1		ALK	2p23.2-p23.1	
t(9;9)(p21;p21)	MTS2	9p21.3	FRA9A (rare, folic acid), FRA9C (common, BrdU)	MTS1	9p21.3	FRA9A (rare, folic acid), FRA9C (common, BrdU)
t(6;7)(q23;q36)	MYB	6q23.3		MNX1	7q36.3	FRA7I (common, apc)
t(8;9)(q24;p13)	MYC	8q24.21		ZBTB5	9p13.2	
t(8;9)(q24;p13)	MYC	8q24.21		ZCCHC7	9p13.2	
t(2;22)(p23;q12)	MYH9	22q12.3		ALK	2p23.2-p23.1	
t(8;17)(p12;q11)	MYO18A	17q11.2		FGFR1	8p12	
t(2;8)(p23;p11.2)	MYST3	8p11.21		ASXL2	2p23.3	
t(8;16)(p11;p13)	MYST3	8p11.21		CREBBP	16p13.3	
t(8;22)(p11;q13)	MYST3	8p11.21		EP300	22q13.2	FRA22A (rare, folic acid)
inv(8)(p11q13)	MYST3	8p11.21		NCOA2	8q13.3	
t(8;20)(p11;q13)	MYST3	8p11.21		NCOA3	20q13.12	
t(10;16)(q22;p13)	MYST4	10q22.2		CREBBP	16p13.3	
inv(10)(q11q11)	NCOA4	10q11.23	FRA10G (common, apc)	RET	10q11.21	FRA10G (common, apc)
t(5;16)(q33;p13)	NDE1	16p13.11	FRA16A (rare, folic acid)	PDGFRB	5q33.1	
del(10)(q24q24)*	NFKB2	10q24.32		INA	10q24.33	
t(7;10)(q34;q24)	NFKB2	10q24.32		TBXAS1	7q34	
t(5;14)(q33;q22)	NIN	14q22.1		PDGFRB	5q33.1	
t(12;19)(p13.3; p13.3)	NOL1	12p13.31		TCF3	19p13.3	FRA19B (rare, folic acid)
inv(X)(p11q13)	NONO	Xq13.1		TFE3	Xp11.23	
t(7;9)(q34;q34)	NOTCH1	9q34.3		TRB@	7q34	
t(2;5)(p23;q35)	NPM1	5q35.1	FRA5G (rare, folic acid)	ALK	2p23.2-p23.1	
t(3;5)(q25;q35)	NPM1	5q35.1	FRA5G (rare, folic acid)	MLF1	3q25.32	FRA3D (common, apc)
t(5;17)(q35;q21)	NPM1	5q35.1	FRA5G (rare, folic acid)	RARA	17q21.2	
t(3;5)(p24;q35)	NSD1	5q35.2-q35.3	FRA5G (rare, folic acid)	ANKRD28	3p24.3	
t(11;17)(q13;q21)	NUMA1	11q13.4	FRA11H (common, apc)	RARA	17q21.2	
t(9;9)(q34;q34)	NUP214	9q34.13		ABL1	9q34.12	
t(6;9)(p22;q34)	NUP214	9q34.13		DEK	6p22.3	
t(10;11)(q25;p15)	NUP98	11p15.4		ADD3	10q25.1-q25.2	FRA10B (rare, BrdU), FRA10E (common apc)
t(6;11)(q24;p15)	NUP98	11p15.4		CCDC28A	6q24.1	
inv(11)(p15q22)	NUP98	11p15.4		DDX10	11q22.3	
t(10;11)(q23;p15)	NUP98	11p15.4		HHEX	10q23.33	FRA10A (rare, folic acid)
t(7;11)(p15;p15)	NUP98	11p15.4		HOXA11	7p15.2	
t(7;11)(p15;p15)	NUP98	11p15.4		HOXA13	7p15.2	
t(7;11)(p15;p15)	NUP98	11p15.4		HOXA9	7p15.2	
t(11;12)(p15;q13)	NUP98	11p15.4		HOXC11	12q13.13	FRA12A (rare, folic acid)
t(11;12)(p15;q13)	NUP98	11p15.4		HOXC13	12q13.13	FRA12A (rare, folic acid)
t(2;11)(q31;p15)	NUP98	11p15.4		HOXD11	2q31.1	FRA2G (common, apc)
t(2;11)(q31;p15)	NUP98	11p15.4		HOXD13	2q31.1	FRA2G (common, apc)
t(3;11)(q29q13;p15)del(3)(q29).	NUP98	11p15.4		IQCG	3q29	
t(5;11)(q35;p15)	NUP98	11p15.4		NSD1	5q35.2-q35.3	FRA5G (rare, folic acid)
t(11;17)(p15;p13)	NUP98	11p15.4		PHF23	17p13.1	
t(1;11)(q24;p15)	NUP98	11p15.4		PRRX1	1q24.2	
t(9;11)(q34;p15)	NUP98	11p15.4		PRRX2	9q34.11	
t(9;11)(p22;p15)	NUP98	11p15.4		PSIP1	9p22.3	
t(4;11)(q23;p15)	NUP98	11p15.4		RAP1GDS1	4q23	

t(11;18)(p15;q12)	NUP98	11p15.4		SETBP1	18q12.3	
t(11;20)(p15;q12)	NUP98	11p15.4		TOP1	20q12	
t(3;11)(p24;p15)	NUP98	11p15.4		TOP2B	3p24.2	FRA3A (common, apc)
t(8;11)(p12;p15)	NUP98	11p15.4		WHSC1L1	8p12	
t(15;19)(q14;p13)	NUT	15q14		BRD4	19q13.12	FRA19B (rare, folic acid)
t(8;11)(p12;q14)	ODZ4	11q14.1		NRG1	8p12	
t(9;17)(q22;p13)	OMD	9q22.31		USP6	17p13.2	
t(2;13)(q36;q14)	PAX3	2q36.1		FOXO1A	13q14.11	
t(X;2)(q13;q36)	PAX3	2q36.1		MLLT7	Xq13.1	
t(2;2)(p23;q36)	PAX3	2q36.1		NCOA1	2p23.3	
t(7;9)(q11;p13)	PAX5	9p13.2		ELN	7q11.23	FRA7J (common, apc)
t(9;12)(p13;p13)	PAX5	9p13.2		ETV6	12p13.2	
t(3;9)(p14;p13)	PAX5	9p13.2		FOXP1	3p14.1	
t(9;15)(p13;q24)	PAX5	9p13.2		PML	15q24.1	
t(9;18)(p13;q11)	PAX5	9p13.2		ZNF521	18q11.2	
t(1;13)(p36;q14)	PAX7	1p36.13	FRA1A (common, apc)	FOXO1A	13q14.11	
t(2;3)(q13;p25)	PAX8	2q13	FRA2B (rare, folic acid)	PPARG	3p25.2-p25.1	
t(8;9)(p22;p24)	PCM1	8p22		JAK2	9p24.1	
t(8;10)(p22;q11)	PCM1	8p22		RET	10q11.21	FRA10G (common, apc)
t(1;5)(q21;q33)	PDE4DIP	1q21.1	FRA1F (common, apc)	PDGFRB	5q33.1	
t(10;11)(p12;q14)	PICALM	11q14.2	FRA11F (common, apc)	MLLT10	10p12.31	
t(3;6)(q27;p21)	PIM1	6p21.2	FRA6H (common, apc)	BCL6	3q27.3	FRA3C (common, apc)
t(15;17)(q24;q21)	PML	15q24.1		RARA	17q21.2	
t(3;11)(q27;q23.1)	POU2AF1	11q23.1		BCL6	3q27.3	FRA3C (common, apc)
t(8;22)(p21;q12)	PPP2R2A	8p21.2		CHEK2	22q12.1	FRA22B (common, apc)
t(X;1)(p11;q23)	PRCC	1q23.1		TFE3	Xp11.23	
t(17;17)(q21;q24)	PRKAR1A	17q24.2		RARA	17q21.2	
t(10;17)(q11;q24)	PRKAR1A	17q24.2		RET	10q11.21	FRA10G (common, apc)
t(4;5)(q21;q33)	PRKG2	4q21.21		PDGFRB	5q33.1	
t(14;19)(q11;q13)	PVRL2	19q13.32	FRA19A (common, 5-aza)	TRA@	14q11.2	
t(5;17)(q33;p13)	RABEP1	17p13.2		PDGFRB	5q33.1	
t(5;14)(q35;q11)	RANBP17	5q35.1	FRA5G (rare, folic acid)	TRD@	14q11.2	
t(2;2)(p23;q13)	RANBP2	2q13	FRA2B (rare, folic acid)	ALK	2p23.2-p23.1	
t(1;22)(p13;q13)	RBM15	1p13.3		MKL1	22q13.1-q13.2	FRA22A (rare, folic acid)
t(3;5)(p21;q33)	RBM6	3p21.31		CSF1R	5q33.1	
t(3;4)(q27;p14)	RHOH	4p14		BCL6	3q27.3	FRA3C (common, apc)
inv(3)(q21q26), t(3;3)(q21;q26)	RPN1	3q21.3		EVI1	3q26.2	
t(1;3)(p36;q21)	RPN1	3q21.3		PRDM16	1p36.32	FRA1A (common, apc)
t(2;21)(q11;q22)	RUNX1	21q22.12		AFF3	2q11.2	FRA2A (rare, folic acid)
t(16;21)(q24;q22)	RUNX1	21q22.12		CBFA2T3	16q24.3	
t(12;21)(q12;q22)	RUNX1	21q22.12		CPNE8	12q12	
t(3;21)(q26;q22)	RUNX1	21q22.12		EVI1	3q26.2	
t(11;21)(q13;q22)	RUNX1	21q22.12		MACROD1	11q13.1	FRA11H (common, apc)
t(3;21)(q26;q22)	RUNX1	21q22.12		MDS1	3q26.2	
t(1;21)(p36;q22)	RUNX1	21q22.12		PRDM16	1p36.32	FRA1A (common, apc)
t(X;21)(p22;q22)	RUNX1	21q22.12		PRDX4	Xp22.11	

t(3;21)(q26;q22)	RUNX1	21q22.12		RPL22P1	3q26.2	
t(8;21)(q21;q22)	RUNX1	21q22.12		RUNX1T1	8q21.3	
t(4;21)(q31;q22)	RUNX1	21q22.12		SH3D19	4q31.3	
t(8;21)(q23;q22)	RUNX1	21q22.12		TRPS1	8q23.3	
t(7;21)(p22;q22)	RUNX1	21q22.12		USP42	7p22.1	FRA7B (common, apc)
t(1;21)(p35;q22)	RUNX1	21q22.12		YTHDF2	1p35.3	
t(1;21)(q21;q22)	RUNX1	21q22.12		ZNF687	1q21.2	FRA1F (common, apc)
t(2;4)(p23;q21)	SEC31A	4q21.22		ALK	2p23.2-p23.1	
del(6)(q14q22)*	SENP6	6q14.1		TCBA1	6q22.31	
t(9;9)(q34;q34), del(9)(q34q34)*	SET	9q34.11		NUP214	9q34.13	
t(1;9)(p34;q34)	SFPQ	1p34.3		ABL1	9q34.12	
t(X;1)(p11;p34)	SFPQ	1p34.3		TFE3	Xp11.23	
t(3;6)(q27;p21)	SFRS3	6p21.31	FRA6H (common, apc)	BCL6	3q27.3	FRA3C (common, apc)
t(4;6)(p15;q22)	SLC34A2	4p15.2	FRA4D (common, apc)	ROS1	6q22.2	
t(1;7)(q32;p21)	SLC45A3	1q32.1		ETV1	7p21.2	
t(1;3)(q32;q27)	SLC45A3	1q32.1		ETV5	3q27.2	FRA3C (common, apc)
t(5;17)(q33;p11.2)	SPECC1	17p11.2		PDGFRB	5q33.1	
t(2;5)(p16;q33)	SPTBN1	2p16.2	FRA2D (common, apc)	PDGFRB	5q33.1	
t(X;18)(p11;q11)	SS18	18q11.2		SSX1	Xp11.23	
t(X;18)(p11;q11)	SS18	18q11.2		SSX2	Xp11.22	
t(X;18)(p11;q11)	SS18	18q11.2		SSX4	Xp11.23	
t(X;20)(p11;q13)	SS18L1	20q13.33		SSX1	Xp11.23	
t(17;17)(q21;q21)	STAT5B	17q21.2		RARA	17q21.2	
del(1)(p33p33)*	STIL	1p33		TAL1	1p33	
t(2;4)(p22;q12)	STRN	2p22.2		PDGFRA	4q12	FRA4B (common, BrdU)
t(9;17)(q31;q12)	TAF15	17q12		NR4A3	9q31.1	
t(12;17)(p13;q12)	TAF15	17q12		ZNF384	12p13.31	
t(1;3)(p32-34;p21)	TAL1	1p33		RHOA	3p21.31	
t(1;7)(p33;q34)	TAL1	1p33		TRB@	7q34	
t(1;14)(p33;q11)	TAL1	1p33		TRD@	14q11.2	
t(3;6)(q26;q25)	TBL1XR1	3q26.32		RGS17	6q25.2	
t(8;8)(q11;q12)	TCEA1	8q11.23		PLAG1	8q12.1	
t(9;15)(q31;q21)	TCF12	15q21.3		NR4A3	9q31.1	
t(17;19)(q22;p13)	TCF3	19p13.3	FRA19B (rare, folic acid)	HLF	17q22	
t(1;19)(q23;p13)	TCF3	19p13.3	FRA19B (rare, folic acid)	PBX1	1q23.3	
t(19;19)(p13;q13)	TCF3	19p13.3	FRA19B (rare, folic acid)	TFPT	19q13.42	FRA19A (common, 5-aza)
t(12;19)(p13;p13)	TCF3	19p13.3	FRA19B (rare, folic acid)	ZNF384	12p13.31	
t(1;3)(p33;p21)	TCTA	3p21.31		TAL1	1p33	
t(2;3)(p23;q12)	TFG	3q12.2		ALK	2p23.2-p23.1	
t(3;9)(q12;q31)	TFG	3q12.2		NR4A3	9q31.1	
t(1;3)(q23;q12)	TFG	3q12.2		NTRK1	1q23.1	
t(3;3)(q29;q27)	TFRC	3q29		BCL6	3q27.3	FRA3C (common, apc)
t(2;3)(p21;q26)	THADA	2p21		MDS1	3q26.2	
t(1;17)(p34;p13)	THRAP3	1p34.3		USP6	17p13.2	
t(7;10)(q34;q24)	TLX1	10q24.31		TRB@	7q34	
t(10;14)(q24;q11)	TLX1	10q24.31		TRD@	14q11.2	

t(21;21)(q22;q22), del(21)(q22q22)*	TMPRSS2	21q22.3		ERG	21q22.2	
t(7;21)(p21;q22)	TMPRSS2	21q22.3		ETV1	7p21.2	
t(17;21)(q21;q22)	TMPRSS2	21q22.3		ETV4	17q21.31	
t(3;21)(q27;q22)	TMPRSS2	21q22.3		ETV5	3q27.2	FRA3C (common, apc)
t(5;15)(q33;q15)	TP53BP1	15q15.3		PDGFRB	5q33.1	
t(1;2)(q21;p23)	TPM3	1q21.3	FRA1F (common, apc)	ALK	2p23.2-p23.1	
inv(1)(q21q23)	TPM3	1q21.3	FRA1F (common, apc)	NTRK1	1q23.1	
t(1;5)(q21;q33)	TPM3	1q21.3	FRA1F (common, apc)	PDGFRB	5q33.1	
inv(1)(q21q31)	TPM3	1q21.3	FRA1F (common, apc)	TPR	1q31.1	FRA1K (common, apc)
t(2;19)(p23;p13)	TPM4	19p13.12	FRA19B (rare, folic acid)	ALK	2p23.2-p23.1	
inv(1)(q23q31)	TPR	1q31.1	FRA1K (common, apc)	NTRK1	1q23.1	
t(9;14)(p21;q11)	TRA@	14q11.2		CDKN2A	9p21.3	FRA9A (rare, folic acid), FRA9C (common, BrdU)
t(X;14)(q28;q11)	TRA@	14q11.2		MTCP1	Xq28	FRAXE (rare, folic acid), FRAXF (rare, folic acid)
t(8;14)(q24;q11)	TRA@	14q11.2		MYC	8q24.21	
t(14;21)(q11;q22)	TRA@	14q11.2		OLIG2	21q22.11	
t(14;14)(q11;q32)	TRA@	14q11.2		TCL1A	14q32.13	
t(7;14)(q34;q11)	TRA@	14q11.2		TRB@	7q34	
t(7;14)(p14;q11)	TRA@	14q11.2		TRG@	7p14.1	
t(7;12)(q34;p13)	TRB@	7q34		CCND2	12p13.32	
inv(7)(p15q34), t(7;7)(p15;q34)	TRB@	7q34		HOX9	7p15.2	
inv(7)(p15q34)	TRB@	7q34		HOXA10	7p15.2	
inv(7)(p15q34)	TRB@	7q34		HOXA11	7p15.2	
t(7;11)(q34;p15)	TRB@	7q34		LMO1	11p15.4	
t(7;11)(q34;p13)	TRB@	7q34		LMO2	11p13	FRA11E (common, apc)
t(X;7)(q28;q34)	TRB@	7q34		MTCP1	Xq28	FRAXE (rare, folic acid), FRAXF (rare, folic acid)
t(6;7)(q23;q34)	TRB@	7q34		MYB	6q23.3	
t(7;9)(q34;q31)	TRB@	7q34		TAL2	9q31.2	
inv(7)(p14q34)	TRB@	7q34		TRG@	7p14.1	
t(11;14)(p15;q11)	TRD@	14q11.2		LMO1	11p15.4	
t(11;14)(p13;q11)	TRD@	14q11.2		LMO2	11p13	FRA11E (common, apc)
t(5;14)(q35;q11)	TRD@	14q11.2		NKX2E	5q35.2	FRA5G (rare, folic acid)
t(8;14)(q24;q11)	TRD@	14q11.2		PVT1	8q24.21	
t(5;14)(q35;q11)	TRD@	14q11.2		TLX3	5q35.1	FRA5G (rare, folic acid)
t(7;14)(p14;q32)	TRG@	7p14.1		IGH@	14q32.33	
t(7;8)(q34;p12)	TRIM24	7q34		FGFR1	8p12	
t(7;10)(q34;q11)	TRIM24	7q34		RET	10q11.21	FRA10G (common, apc)
t(1;10)(p13;q11)	TRIM33	1p13.2		RET	10q11.21	FRA10G (common, apc)
t(5;14)(q33;q32)	TRIP11	14q32.12		PDGFRB	5q33.1	
t(12;13)(p13;q14)	TTL-T	13q14.11		ETV6	12p13.2	
t(11;17)(q23;q21)	ZBTB16	11q23.2		RARA	17q21.2	
t(9;10)(q34;q22.3)	ZMIZ1	10q22.3		ABL1	9q34.12	
t(8;13)(p12;q12)	ZMYM2	13q12.11		FGFR1	8p12	
t(3;7)(q27;p12)	ZNFN1A1	7p12.2		BCL6	3q27.3	FRA3C (common, apc)
