# Supplementary material for: Over half of breakpoints in gene pairs involved in cancer-specific recurrent translocations are mapped to human chromosomal fragile sites
Source: BMC Genomics. 2009 Jan 30;10:59. doi: 10.1186/1471-2164-10-59 (PMC2642838; doi:10.1186/1471-2164-10-59)
Supplement: Additional file 2 — Gene pairs involved in cancer-specific recurrent translocations in which the breakpoint in one gene co-localizes with a fragile site. The translocation name(s) for each unique gene set is indicated. The gene within each set located at a fragile site is highlighted in gray, and the cancer(s) in which the fusion transcript is found is included. [file 1471-2164-10-59-S2.pdf]

Additional file 2 - Gene pairs involved in cancer-specific recurrent translocations in which the breakpoint in one gene co-localizes with a fragile site					
**Translocation**	**Gene**	**Fragile Site**	**Gene**	**Fragile Site**	**Cancer[a]**
t(7;12)(p22;q13)	ACTB	FRA7B	GLI1		Vascular and perivascular tumor
inv(7)(q21q34)	AKAP9	FRA7E	BRAF		Adenocarcinoma (Thyroid)
t(5;14)(q35;q32)	BCL11B		NKX2E	FRA5G	Acute lymphoblastic leukemia/lymphoblastic lymphoma
t(5;14)(q35;q32)	BCL11B		TLX3	FRA5G	Acute lymphoblastic leukemia/lymphoblastic lymphoma
t(14;18)(q32;q21)	BCL2	FRA18B	IGH@		Chronic lymphocytic leukemia, B-cell lymphoma
t(18;22)(q21;q11)	BCL2	FRA18B	IGL@		Chronic lymphocytic leukemia, Mature B-cell neoplasm
t(8;19)(q24;q13)	BCL3	FRA19A	MYC		B-prolymphocytic leukemia
t(3;16)(q27;p13)	BCL6	FRA3C	CIITA		Diffuse large B-cell lymphoma
t(3;8)(q27;q24)	BCL6	FRA3C	MYC		Diffuse large B-cell lymphoma
t(1;14)(q21;q32)	BCL9	FRA1F	IGH@		Acute lymphoblastic leukemia
t(1;22)(q21;q11)	BCL9	FRA1F	IGL@		Follicular lymphoma
t(4;22)(q12;q11)	BCR		PDGFRA	FRA4B	Atypical chronic myeloid leukemia
t(11;18)(q22;q21)	BIRC3		MALT1	FRA18B	B-cell lymphoma
t(8;12)(q24;q21-22)	BTG1	FRA12B	MYC		Chronic lymphocytic leukemia
t(5;10)(q33;q21)	CCDC6	FRA10C	PDGFRB		Chronic myeloid leukemia
t(5;11)(q12;q23)	CENPK		MLL	FRA11B, FRA11G	Acute myeloid leukemia
t(4;12)(q12;p13)	CHIC2	FRA4B	ETV6		Aggressive NK-cell leukemia, Acute myeloid leukemia
t(4;19)(q35;q13)	CIC	FRA19A	DUX4		Soft tissue tumor
t(2;17)(p23;q23)	CLTC	FRA17B	ALK		Inflammatory myofibroblastic tumor, B-cell lymphoma
t(X;17)(p11;q23)	CLTC	FRA17B	TFE3		Adenocarcinoma (Kidney)
t(17;22)(q21;q13)	COL1A1		PDGFB	FRA22A	Dermatofibrosarcoma protuberans/Bednar tumor
t(X;6)(q22;q13-14)	COL4A5		COL12A1	FRA6D	Bone- and cartilage-producing tumor
t(1;2)(p13;q37)	COL6A3	FRA2J	CSF1		Tenosynovial giant cell tumor
t(11;19)(q21;p13)	CRTC1	FRA19B	MAML2		Benign epithelial tumor, special type, Mucoepidermoid carcinoma
t(3;9)(q27;p24)	DMRT1		BCL6	FRA3C	Diffuse large B-cell lymphoma
t(1;1)(p36;q41)	DUSP10		PRDM16	FRA1A	Acute myeloid leukemia
t(10;12)(q11;p13)	ERC1		RET	FRA10G	Papillary Thyroid Carcinoma
t(5;12)(q31;p13)	ETV6		ACSL6	FRA5C	Atypical chronic myeloid leukemia, Acute myeloid leukemia
t(1;12)(q21;p13)	ETV6		ARNT	FRA1F	Acute myeloid leukemia
t(10;12)(q24;p13)	ETV6		GOT1	FRA10A	Myelodysplastic syndrome
t(1;12)(p36;p13)	ETV6		MDS2	FRA1A	Acute myeloid leukemia
t(4;12)(q12;p13)	ETV6		PDGFRA	FRA4B	Chronic eosinophilic leukemia/hypereosinophilic syndrome
t(12;22)(q13;q12)	EWSR1	FRA22B	DDIT3		Myxoid liposarcoma
t(21;22)(q22;q12)	EWSR1	FRA22B	ERG		Ewing tumor/peripheral primitive neuroectodermal tumor
t(7;22)(p21;q12)	EWSR1	FRA22B	ETV1		Ewing tumor/peripheral primitive neuroectodermal tumor
t(17;22)(q21;q12)	EWSR1	FRA22B	ETV4		Ewing tumor/peripheral primitive neuroectodermal tumor
t(2;22)(q35;q12)	EWSR1	FRA22B	FEV		Ewing tumor/peripheral primitive neuroectodermal tumor
t(11;22)(q24;q12)	EWSR1	FRA22B	FLI1		Ewing tumor/peripheral primitive neuroectodermal tumor
t(9;22)(q31;q12)	EWSR1	FRA22B	NR4A3		Chondrosarcoma, myxoid (Soft tissue)
t(12;22)(p13;q12)	EWSR1	FRA22B	ZNF384		Acute undifferentiated leukemia, Acute lymphoblastic leukemia
t(4;17)(q12;q21)	FIP1L1	FRA4B	RARA		Juvenile myelomonocytic leukemia

t(X;11)(q13;q23)	FOXO4		MLL	FRA11B, FRA11G	Acute lymphoblastic leukemia, Acute myeloid leukemia
t(12;16)(q13;p11)	FUS		ATF1	FRA12A	Angiomatoid malignant fibrous histiocytoma (Soft tissue)
t(3;12)(q27;p13)	GAPDH		BCL6	FRA3C	Diffuse large B-cell lymphoma
t(5;12)(q33;q24)	GIT2	FRA12E	PDGFRB		Chronic myeloid proliferative disorders
t(10;14)(q11;q32)	GOLGA5		RET	FRA10G	Papillary Thyroid Carcinoma
del(8)(q12q24)*	HAS2	FRA8C, FRA8E	PLAG1		Lipoblastoma (Soft tissue)
t(8;19)(p12;q13)	HERV-K (LOC113386)	FRA19A	FGFR1		Chronic myeloproliferative disorder
t(5;7)(q33;q11)	HIP1	FRA7J	PDGFRB		Chronic myelomonocytic leukemia
t(3;6)(q27;p22)	HIST1H4I		BCL6	FRA3C	Non-Hodgkin's lymphoma
t(2;12)(q37;q14)	HMGA2		CXCR7	FRA2J	Lipoma (Soft tissue)
t(12;14)(q14;q24)	HMGA2		RAD51L1	FRA14C	Leiomyoma (Uterus, corpus)
t(8;10)(p11;q11)	HOOK3		RET	FRA10G	Papillary Thyroid Carcinoma
t(6;16)(p21;q22)	HP		MRPS10	FRA6H	Prostate carcinoma
t(3;14)(q27;q32)	HSP90AA1		BCL6	FRA3C	Diffuse large B-cell lymphoma
t(1;14)(p22;q32)	IGH@		BCL10	FRA1D	Extranodal marginal zone B-cell lymphoma
t(14;19)(q32;q13)	IGH@		BCL3	FRA19A	B-cell lymphoma
t(3;14)(q27;q32)	IGH@		BCL6	FRA3C	Follicular lymphoma, B-cell lymphoma,
t(11;14)(q13;q32)	IGH@		CCND1	FRA11A, FRA11H	Chronic lymphocytic leukemia
t(6;14)(p21;q32)	IGH@		CCND3	FRA6H	B-cell lymphoma
t(7;14)(q21;q32)	IGH@		CDK6	FRA7E	Chronic lymphocytic leukemia
t(14;19)(q32;q13)	IGH@		CEBPA	FRA19A	Acute lymphoblastic leukemia
t(14;19)(q32;q13)	IGH@		CEBPG	FRA19A	Acute lymphoblastic leukemia
t(11;14)(q23;q32)	IGH@		DDX6	FRA11B, FRA11G	Acute lymphoblastic leukemia
t(7;14)(q21;q32)	IGH@		ERVWE1	FRA7E	Chronic lymphocytic leukemia
t(5;14)(q31;q32)	IGH@		IL3	FRA5C	Acute lymphoblastic leukemia
t(14;18)(q32;q21)	IGH@		MALT1	FRA18B	B-cell lymphoma
t(11;14)(q23;q32)	IGH@		PAFAH1B2	FRA11B, FRA11G	Non-Hodgkin's lymphoma
t(11;14)(q23;q32)	IGH@		PCSK7	FRA11B, FRA11G	Mature B-cell neoplasm
t(14;19)(q32;q13)	IGH@		SPIB	FRA19A	Diffuse large B-cell lymphoma
t(14;16)(q32;q23)	IGH@		WWOX	FRA16D	Multiple myeloma
t(2;12)(p11;p13)	IGK@	FRA2L	CCND2		Mantle cell lymphoma
t(2;8)(p11;q24)	IGK@	FRA2L	MYC		B-cell lymphoma
t(2;8)(p11;q24)	IGK@	FRA2L	PVT1		Burkitt lymphoma/leukemia
t(2;6)(p11;q25)	IGK@	FRA2L	ZC3H12D		Diffuse large B-cell lymphoma
t(19;22)(q13;q11)	IGL@		BCL3	FRA19A	Follicular lymphoma, Diffuse large B-cell lymphoma
t(3;22)(q27;q11)	IGL@		BCL6	FRA3C	B-cell lymphoma
t(11;22)(q13;q11)	IGL@		CCND1	FRA11A, FRA11H	Mature B-cell neoplasm, Mantle cell lymphoma
t(6;22)(p21;q11)	IGL@		CCND3	FRA6H	Multiple myeloma
t(7;22)(q21;q11)	IGL@		CDK6	FRA7E	Chronic lymphocytic leukemia
t(16;22)(q23;q11)	IGL@		WWOX	FRA16D	Multiple myeloma
t(6;7)(p21;p15)	JAZF1		PHF1	FRA6H	Endometrial stromal sarcoma (Uterus, corpus)
t(4;10)(q12;p11)	KIF5B		PDGFRA	FRA4B	Hypereosinophilia
t(10;14)(q11;q22)	KTN1		RET	FRA10G	Papillary Thyroid Carcinoma

t(12;16)(p13;p13)	LAG3		MYH11	FRA16A	Acute myeloid leukemia
t(3;13)(q27;q14)	LCP1		BCL6	FRA3C	Follicular lymphoma, Diffuse large B-cell lymphoma
t(5;8)(p13;q12)	LIFR	FRA5A	PLAG1		Adenoma (Salivary gland)
del(3)(q27q28)*	LPP		BCL6	FRA3C	Diffuse large B-cell lymphoma
t(7;19)(q34;p13)	LYL1	FRA19B	TRB@		Acute lymphoblastic leukemia
t(3;18)(p21;q21)	MALT1	FRA18B	MAP4		Diffuse large B-cell lymphoma
t(1;19)(q23;p13)	MEF2D		DAZAP1	FRA19B	Acute lymphoblastic leukemia
t(10;11)(p12;q23)	MLL	FRA11B, FRA11G	ABI1		Acute myeloid leukemia
t(11;17)(q23;q12)	MLL	FRA11B, FRA11G	ACACA		Acute myeloid leukemia
t(5;11)(q31;q23)	MLL	FRA11B, FRA11G	ARHGAP26		Acute myeloid leukemia
t(11;15)(q23;q15)	MLL	FRA11B, FRA11G	CASC5		Acute myeloid leukemia
t(11;12)(q23;q13)	MLL	FRA11B, FRA11G	CIP29		Acute myeloid leukemia
t(11;16)(q23;p13.3)	MLL	FRA11B, FRA11G	CREBBP		Acute myeloid leukemia
t(9;11)(q33;q23)	MLL	FRA11B, FRA11G	DAB2IP		Acute myeloid leukemia
t(3;11)(q21;q23)	MLL	FRA11B, FRA11G	EEFSEC		Acute lymphoblastic leukemia
t(4;11)(p12;q23)	MLL	FRA11B, FRA11G	FRYL		Acute lymphoblastic leukemia, Acute myeloid leukemia
t(11;17)(q23;p13)	MLL	FRA11B, FRA11G	GAS7		Acute lymphoblastic leukemia, Acute myeloid leukemia
t(11;17)(q23;q12)	MLL	FRA11B, FRA11G	LASP1		Acute myeloid leukemia
t(3;11)(q28;q23)	MLL	FRA11B, FRA11G	LPP		Acute myeloid leukemia
inv(11)(q21q23)	MLL	FRA11B, FRA11G	MAML2		Acute myeloid leukemia
t(11;20)(q23;q11)	MLL	FRA11B, FRA11G	MAPRE1		Acute lymphoblastic leukemia
t(10;11)(p12;q23)	MLL	FRA11B, FRA11G	MLLT10		Acute myeloid leukemia
t(6;11)(q27;q23)	MLL	FRA11B, FRA11G	MLLT4		Acute myeloid leukemia
t(11;17)(q23;q12)	MLL	FRA11B, FRA11G	MLLT6		Acute myeloid leukemia
t(3;11)(p21;q23)	MLL	FRA11B, FRA11G	NCKIPSD		Acute myeloid leukemia
t(11;17)(q23;q21)	MLL	FRA11B, FRA11G	RARA		Acute myeloid leukemia
t(4;11)(q21;q23)	MLL	FRA11B, FRA11G	SEPT11		Chronic neutrophilic leukemia
t(11;22)(q23;q11)	MLL	FRA11B, FRA11G	SEPT5		Acute myeloid leukemia
t(X;11)(q24;q23)	MLL	FRA11B, FRA11G	SEPT6		Acute myeloid leukemia
t(11;17)(q23;q25)	MLL	FRA11B, FRA11G	SEPT9		Acute myeloid leukemia
t(4;11)(q35;q23)	MLL	FRA11B, FRA11G	SORBS2		Acute myeloid leukemia
t(11;15)(q23;q15)	MLL	FRA11B, FRA11G	ZFYVE19		Acute myeloid leukemia
t(7;12)(q36;p13)	MNX1	FRA7I	ETV6		Acute myeloid leukemia
t(6;7)(q23;q36)	MYB		MNX1	FRA7I	Acute myeloid leukemia
t(8;22)(p11;q13)	MYST3		EP300	FRA22A	Acute myeloid leukemia
t(5;16)(q33;p13)	NDE1	FRA16A	PDGFRB		Chronic myelomonocytic leukemia
t(12;19)(p13.3; p13.3)	NOL1		TCF3	FRA19B	Acute leukemia
t(2;5)(p23;q35)	NPM1	FRA5G	ALK		B-cell lymphoma
t(5;17)(q35;q21)	NPM1	FRA5G	RARA		Acute myeloid leukemia
t(3;5)(p24;q35)	NSD1	FRA5G	ANKRD28		Adult myeloid leukemia
t(11;17)(q13;q21)	NUMA1	FRA11H	RARA		Acute myeloid leukemia
t(10;11)(q25;p15)	NUP98		ADD3	FRA10B, FRA10E	Acute lymphoblastic leukemia, Acute myeloid leukemia
t(10;11)(q23;p15)	NUP98		HHEX	FRA10A	Acute myeloid Leukemia

t(11;12)(p15;q13)	NUP98		HOXC11	FRA12A	Acute myeloblastic leukemia with maturation
t(11;12)(p15;q13)	NUP98		HOXC13	FRA12A	Myeloblastic leukemia with maturation
t(2;11)(q31;p15)	NUP98		HOXD11	FRA2G	Acute myeloid leukemia
t(2;11)(q31;p15)	NUP98		HOXD13	FRA2G	Acute myeloid leukemia, Chronic myeloid leukemia
t(5;11)(q35;p15)	NUP98		NSD1	FRA5G	Acute myeloid leukemia
t(3;11)(p24;p15)	NUP98		TOP2B	FRA3A	Acute myeloid leukemia
t(15;19)(q14;p13)	NUT		BRD4	FRA19B	Squamous cell carcinoma
t(7;9)(q11;p13)	PAX5		ELN	FRA7J	Acute lymphoblastic leukemia
t(1;13)(p36;q14)	PAX7	FRA1A	FOXO1A		Alveolar rhabdomyosarcoma (Soft tissue)
t(2;3)(q13;p25)	PAX8	FRA2B	PPARG		Adenoma (Thyroid), Adenocarcinoma (Thyroid)
t(8;10)(p22;q11)	PCM1		RET	FRA10G	Papillary Thyroid Carcinoma
t(1;5)(q21;q33)	PDE4DIP	FRA1F	PDGFRB		Myeloproliferative disorder associated with eosinophilia
t(10;11)(p12;q14)	PICALM	FRA11F	MLLT10		Acute myeloid leukemia
t(3;11)(q27;q23.1)	POU2AF1		BCL6	FRA3C	Acute lymphoblastic leukemia/lymphoblastic lymphoma
t(10;17)(q11;q24)	PRKAR1A		RET	FRA10G	Papillary thyroid carcinoma
t(14;19)(q11;q13)	PVRL2	FRA19A	TRA@		Peripheral T-cell lymphoma, Angioimmunoblastic T-cell lymphoma
t(5;14)(q35;q11)	RANBP17	FRA5G	TRD@		Acute lymphoblastic leukemia/lymphoblastic lymphoma
t(2;2)(p23;q13)	RANBP2	FRA2B	ALK		Inflammatory myofibroblastic tumor
t(1;22)(p13;q13)	RBM15		MKL1	FRA22A	Acute myeloid leukemia
t(3;4)(q27;p14)	RHOH		BCL6	FRA3C	Follicular lymphoma, Mature B-cell neoplasm
t(1;3)(p36;q21)	RPN1		PRDM16	FRA1A	Acute myeloid leukemia, Chronic myelomonocytic leukemia
t(2;21)(q11;q22)	RUNX1		AFF3	FRA2A	Childhood T-cell acute lymphblastic leukemia
t(11;21)(q13;q22)	RUNX1		MACROD1	FRA11H	Acute myeloid leukemia,
t(1;21)(p36;q22)	RUNX1		PRDM16	FRA1A	Acute myeloid leukemia
t(7;21)(p22;q22)	RUNX1		USP42	FRA7B	Acute myeloid leukemia
t(1;21)(q21;q22)	RUNX1		ZNF687	FRA1F	Acute myeloid leukemia
t(4;6)(p15;q22)	SLC34A2	FRA4D	ROS1		Carcinoma, NOS (Lung)
t(1;3)(q32;q27)	SLC45A3		ETV5	FRA3C	Adenocarcinoma (Prostate)
t(2;5)(p16;q33)	SPTBN1	FRA2D	PDGFRB		Chronic myeloproliferative disorder,
t(2;4)(p22;q12)	STRN		PDGFRA	FRA4B	Chronic eosinophilic leukemia/hypereosinophilic syndrome
t(17;19)(q22;p13)	TCF3	FRA19B	HLF		Acute lymphoblastic leukemia
t(1;19)(q23;p13)	TCF3	FRA19B	PBX1		Acute lymphoblastic leukemia, Acute myeloid leukemia
t(12;19)(p13;p13)	TCF3	FRA19B	ZNF384		Acute lymphoblastic leukemia
t(3;3)(q29;q27)	TFRC		BCL6	FRA3C	Diffuse large B-cell lymphoma
t(3;21)(q27;q22)	TMPRSS2		ETV5	FRA3C	Adenocarcinoma (Prostate)
t(1;2)(q21;p23)	TPM3	FRA1F	ALK		Anaplastic large cell lymphoma, Inflammatory myofibroblastic tumor
inv(1)(q21q23)	TPM3	FRA1F	NTRK1		Papillary thyroid carcinoma
t(1;5)(q21;q33)	TPM3	FRA1F	PDGFRB		Chronic eosinophilic leukemia
t(2;19)(p23;p13)	TPM4	FRA19B	ALK		Inflammatory myofibroblastic tumor, Anaplastic large cell lymphoma
inv(1)(q23q31)	TPR	FRA1K	NTRK1		Papillary thyroid carcinoma
t(9;14)(p21;q11)	TRA@		CDKN2A	FRA9A, FRA9C	Acute lymphoblastic leukemia
t(X;14)(q28;q11)	TRA@		MTCP1	FRAXE, FRAXF	T-prolymphocytic leukemia, Nonneoplastic lymphatic disorder/lesion

t(7;11)(q34;p13)	TRB@		LMO2	FRA11E	Acute lymphoblastic leukemia
t(X;7)(q28;q34)	TRB@		MTCP1	FRAXE, FRAXF	T-prolymphocytic leukemia
t(11;14)(p13;q11)	TRD@		LMO2	FRA11E	Acute lymphoblastic leukemia
t(5;14)(q35;q11)	TRD@		NKX2E	FRA5G	Acute lymphoblastic leukemia/lymphoblastic lymphoma
t(5;14)(q35;q11)	TRD@		TLX3	FRA5G	Acute lymphoblastic leukemia
t(7;10)(q34;q11)	TRIM24		RET	FRA10G	Papillary thyroid carcinoma
t(1;10)(p13;q11)	TRIM33		RET	FRA10G	Papillary Thyroid Carcinoma
t(3;7)(q27;p12)	ZNFN1A1		BCL6	FRA3C	Diffuse large B-cell lymphoma
a. For a complete description, see the Mitelman database of Chromosome Aberrations in Cancer [27].					
