# Supplementary figures and images for: Over half of breakpoints in gene pairs involved in cancer-specific recurrent translocations are mapped to human chromosomal fragile sites
Source: BMC Genomics. 2009 Jan 30;10:59. doi: 10.1186/1471-2164-10-59 (PMC2642838; doi:10.1186/1471-2164-10-59)

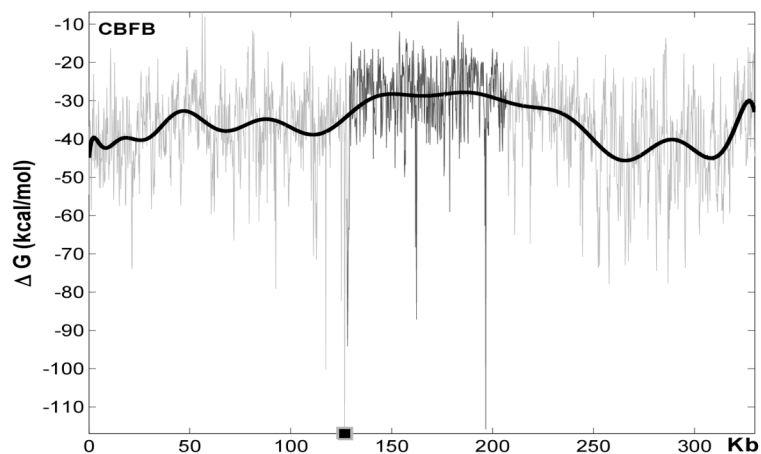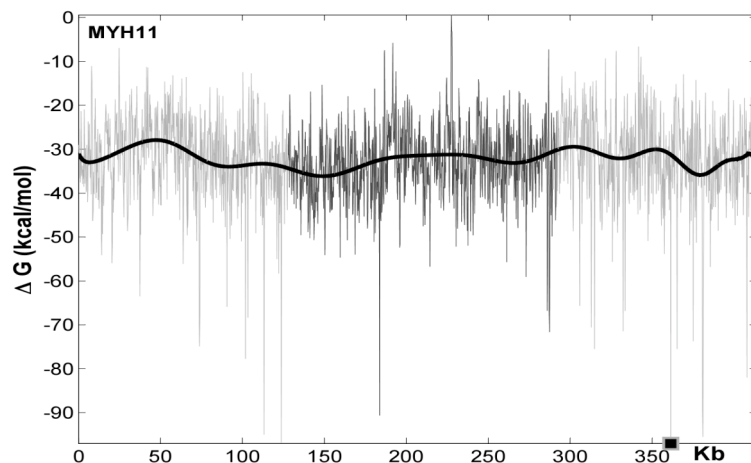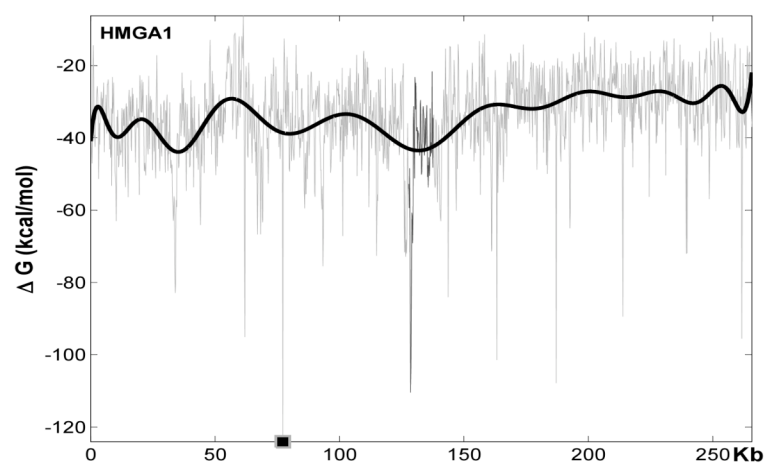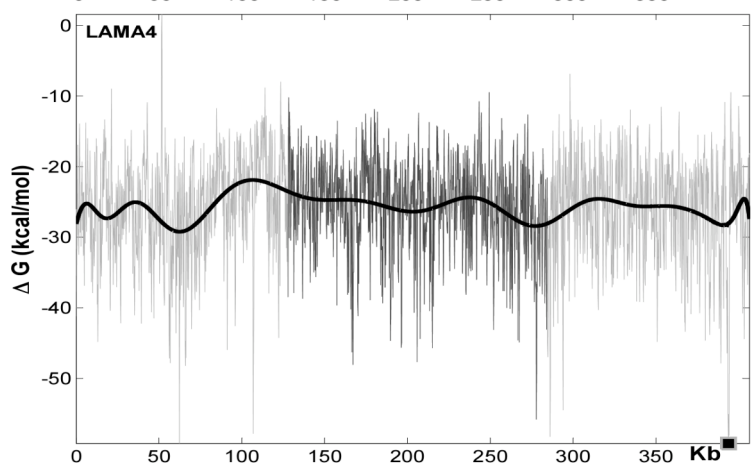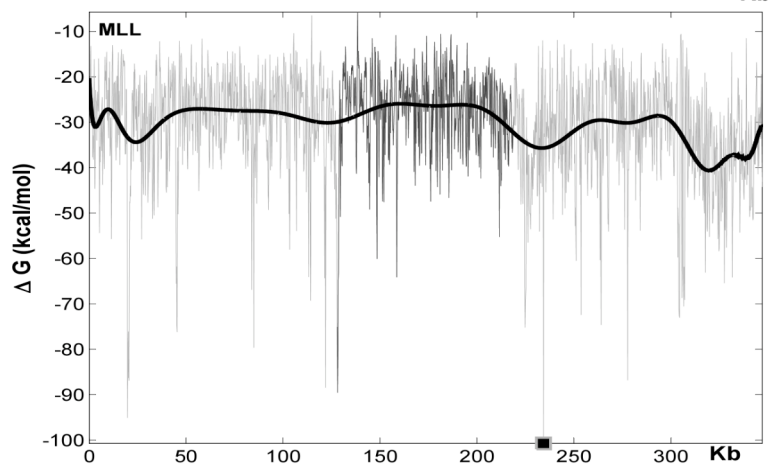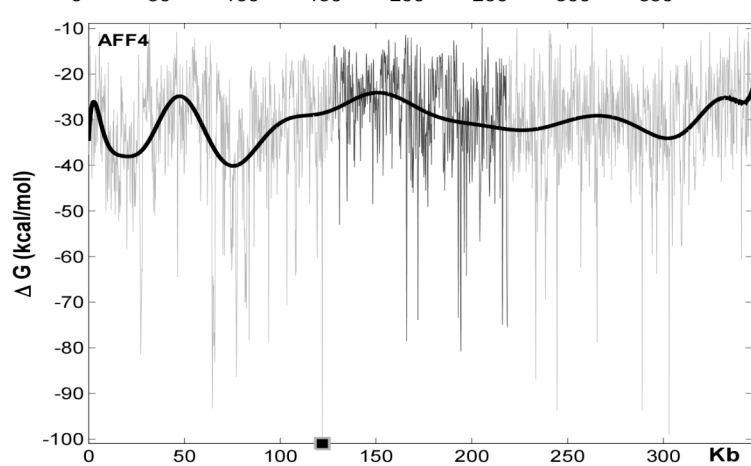

Supplement: Additional file 3 — The computed lowest free energy of predicted DNA secondary structures. The flanking 125 kb regions are shaded in light gray, and the gene region is shaded in black. The black box indicates the location of the most stable structure found in the sequence. The black line is the curve which best fits the raw data. These curves were generated using the polyfit function of the Matlab program, and are presented in Figure 2A. [file 1471-2164-10-59-S3.pdf]
